# Supplementary figures and images for: Proteomic and phosphoproteomic analyses reveal that TORC1 is reactivated by pheromone signaling during sexual reproduction in fission yeast
Source: PLoS Biol. 2024 Dec 20;22(12):e3002963. doi: 10.1371/journal.pbio.3002963 (PMC11750111; doi:10.1371/journal.pbio.3002963)

Bérard, Figure S1

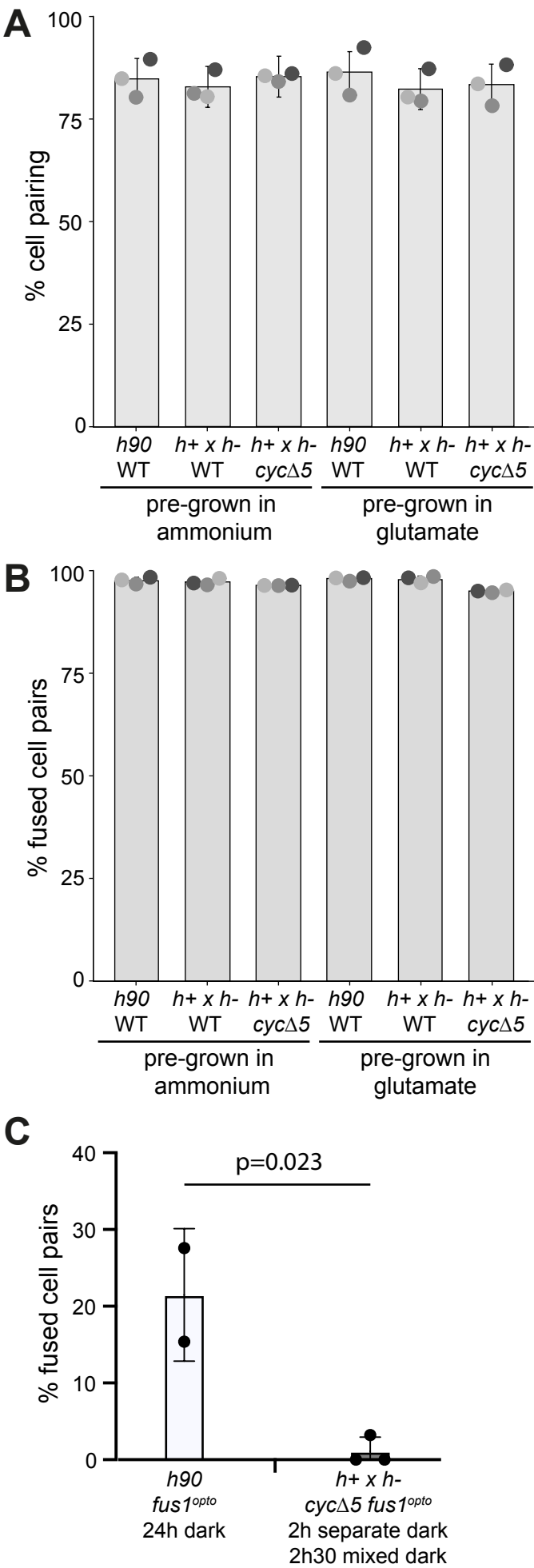

Supplement: S1 Fig — (A, B) Mating efficiency (% of cell pairs among all cells; A) and fusion efficiency (% of paired cells that fused; B) of WT or cycΔ5 cells after 24 h on plates lacking nitrogen. The cells were pre-grown on ammonium or glutamate-containing medium. (C) Fusion efficiency (% of paired cells that fused) of h90 fus1opto cells starved and kept in the dark for 24 h (same data as on Fig 1D) and of h+ x h- cycΔ5 fus1opto cells individually starved for 2 h and then mixed and kept in the dark for a further 2 h 30. The latter h+ x h- cycΔ5 fus1opto conditions are identical to those used in the (phospho)proteomic time course, corresponding to time 150 min in Fig 3A. As these cells do not fuse, this confirms that all phosphorylation changes observed in the mating time course occur before gamete fusion. T test p-value is indicated. The underlying data can be found in S1 Data. (PDF) [file pbio.3002963.s001.pdf]

Bérard, Figure S7

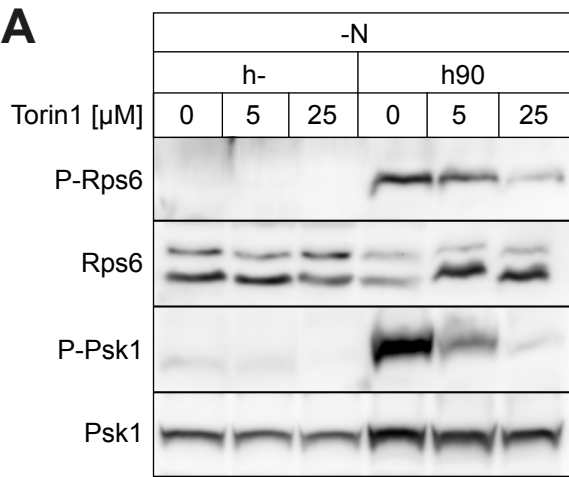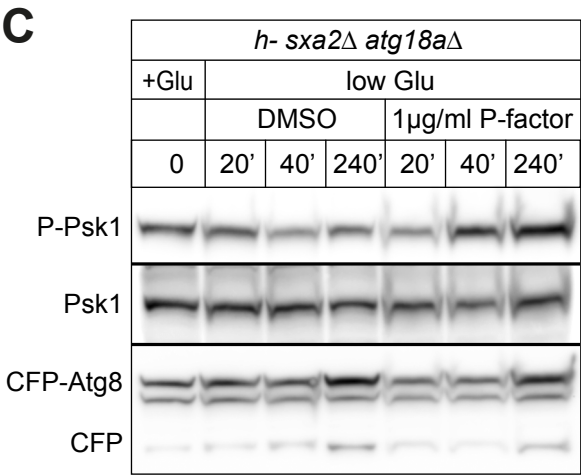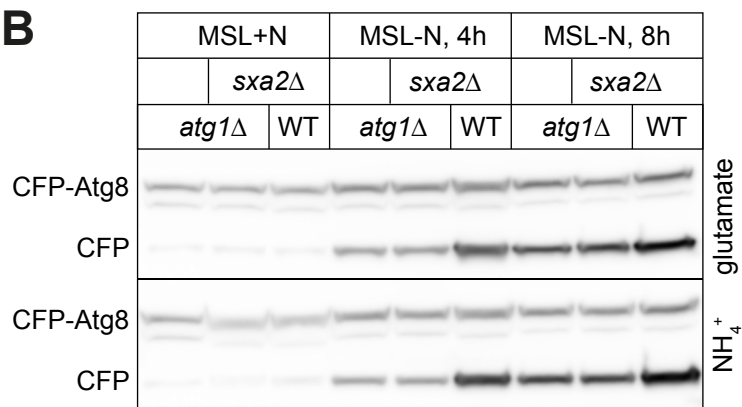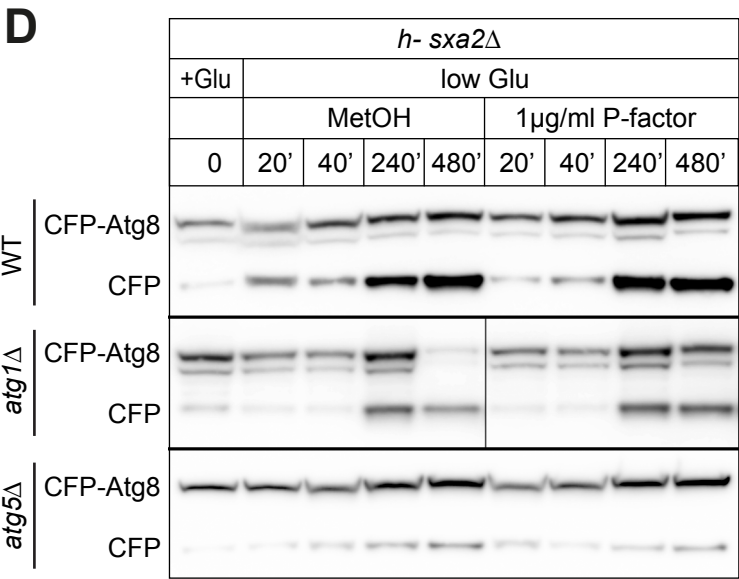

Supplement: S7 Fig — (A) Phospho-Rps6 and phospho-Psk1 levels in WT heterothallic (h-) and homothallic (h90) cells starved for 6 h 15 and then treated with Torin1 (5 or 25 μm) or DMSO (0) for 30 min. (B) CFP-Atg8 cleavage in WT and atg1Δ mutants after 4 h and 8 h of nitrogen starvation in MSL. The cells were pre-grown in MSL + glutamate (top) or MSL + ammonium (bottom). (C) Time course of phospho-Psk1 in h- sxa2Δ atg18aΔ cells transferred to MSL + 0.75 mg/ml glutamate in the presence of P-factor (1 μg/ml) or MetOH at T = 0 min. CFP-Atg8 cleavage is shown in the bottom blot. (D) Time course of CFP-Atg8 cleavage in h- sxa2Δ cells, which are otherwise WT (top), atg1Δ (middle), or atg5Δ (bottom), transferred to MSL + 0.75 mg/ml (or 0.5 mg/ml in case of atg1Δ) glutamate in the presence of P-factor (1 μg/ml) or MetOH at T = 0 min. These are the same extracts as probed for phospho-Psk1 in Fig 6F. Uncropped western blots available in S1 Raw Images. (PDF) [file pbio.3002963.s007.pdf]
