# Supplementary material for: Proteomic and phosphoproteomic analyses reveal that TORC1 is reactivated by pheromone signaling during sexual reproduction in fission yeast
Source: PLoS Biol. 2024 Dec 20;22(12):e3002963. doi: 10.1371/journal.pbio.3002963 (PMC11750111; doi:10.1371/journal.pbio.3002963)
Supplement: S2 Fig — (A) Example profiles of phosphosites in the 3 biological replicates of the starvation/mating time course and the 4 biological replicates of the cell–cell fusion time course. Starvation (cells plated on distinct plates), mating (h+ and h- partners plated together), and starvation-corrected mating values (mating—starvation) are shown in the first 3 columns. The cell–cell fusion time course is shown in the last column. The first 3 examples (Spk1, Rps601, and Rec10) show examples of sites that do not vary significantly upon starvation but change during mating and cell–cell fusion. The next example (Ght1) shows a site that varies during starvation similarly whether cells are starved separately or in presence of their mating partner, resulting in absence of change in the starvation-corrected (mating–starvation) values, indicating no specific change during mating. The last 2 examples show sites that vary significantly both during starvation and mating, but with distinct dynamics. In the Mei2 example, variation occurs in opposite direction. In the Fur4 example, the slope of the change is distinct. Red line indicates average; black lines indicate individual replicates. Pale graphs indicate absence of significant change. The underlying data can be found in S1 and S2 Tables. (B) Short list of expected and identified phosphorylation increases during mating. References are to the expected sites. (PDF) [file pbio.3002963.s002.pdf]

Bérard, Figure S2

A

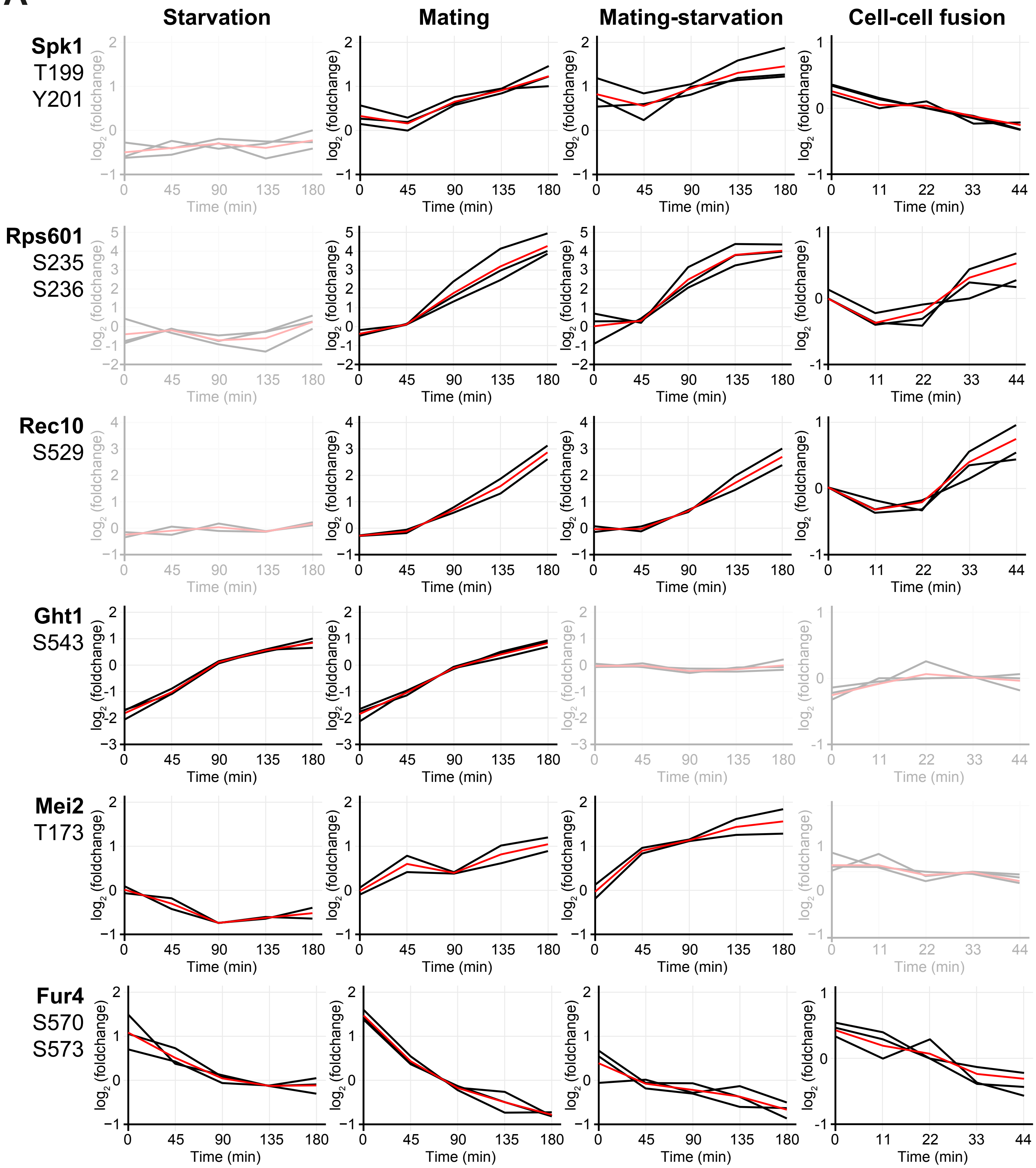

B

Protein	Identified phosphorylation	Expected phosphorylation	Reference
Spk1	T199, Y201	T199, Y201 (active site)	Kelsall et al, 2019
Mam2	T325		
Map3	S346, S349	S/T in C-terminal tail	Hirota et al, 2001
Ste11	T173 (Pat1 target)	T305, T317 (Spk1 targets)	Kjaerulff et al, 2005
