# Supplementary material for: Proteomic and phosphoproteomic analyses reveal that TORC1 is reactivated by pheromone signaling during sexual reproduction in fission yeast
Source: PLoS Biol. 2024 Dec 20;22(12):e3002963. doi: 10.1371/journal.pbio.3002963 (PMC11750111; doi:10.1371/journal.pbio.3002963)

# Bérard, Figure S3

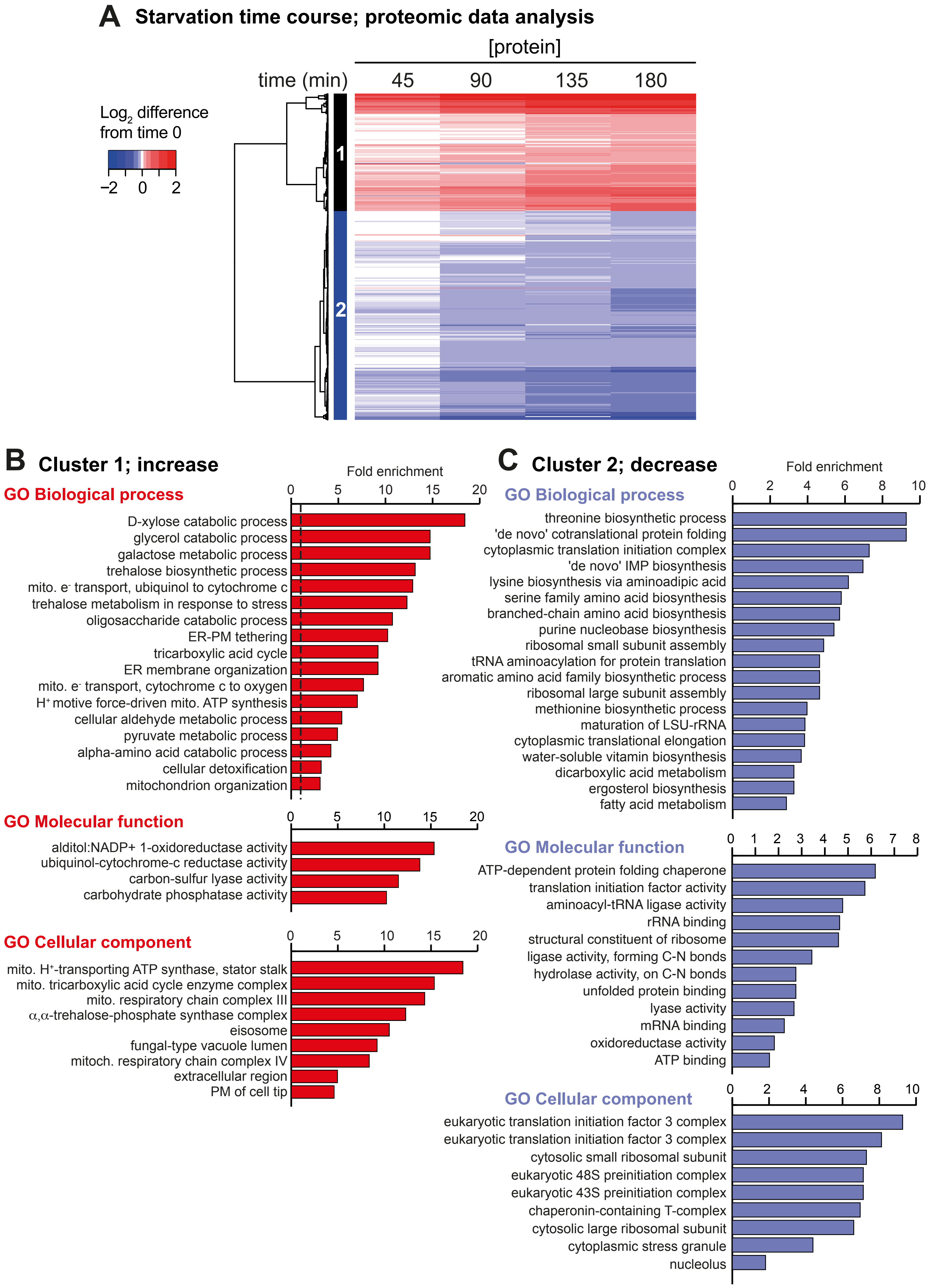

Supplement: S3 Fig — Changes in the proteome of heterothallic cells in a time course of nitrogen starvation starting at t0 = plating of cells on MSL-N plates 2 h after transfer to liquid MSL-N. (A) Heatmap of the significant changes in the levels of 847 proteins during nitrogen starvation, showing 2 major clusters of proteins whose level increase (1) or decrease (2). The underlying data can be found in S1 Table. (B) Significant fold enrichment in GO annotations for biological processes, molecular functions, and cellular components of proteins whose level increases during nitrogen starvation. (C) Significant fold enrichment in GO annotations for biological processes, molecular functions, and cellular components of proteins whose level decreases during nitrogen starvation. Significance levels were assessed by Fisher’s exact test and corrected for false discovery rate. (PDF) [file pbio.3002963.s003.pdf]
