# Supplementary material for: Proteomic and phosphoproteomic analyses reveal that TORC1 is reactivated by pheromone signaling during sexual reproduction in fission yeast
Source: PLoS Biol. 2024 Dec 20;22(12):e3002963. doi: 10.1371/journal.pbio.3002963 (PMC11750111; doi:10.1371/journal.pbio.3002963)

Bérard, Figure S4

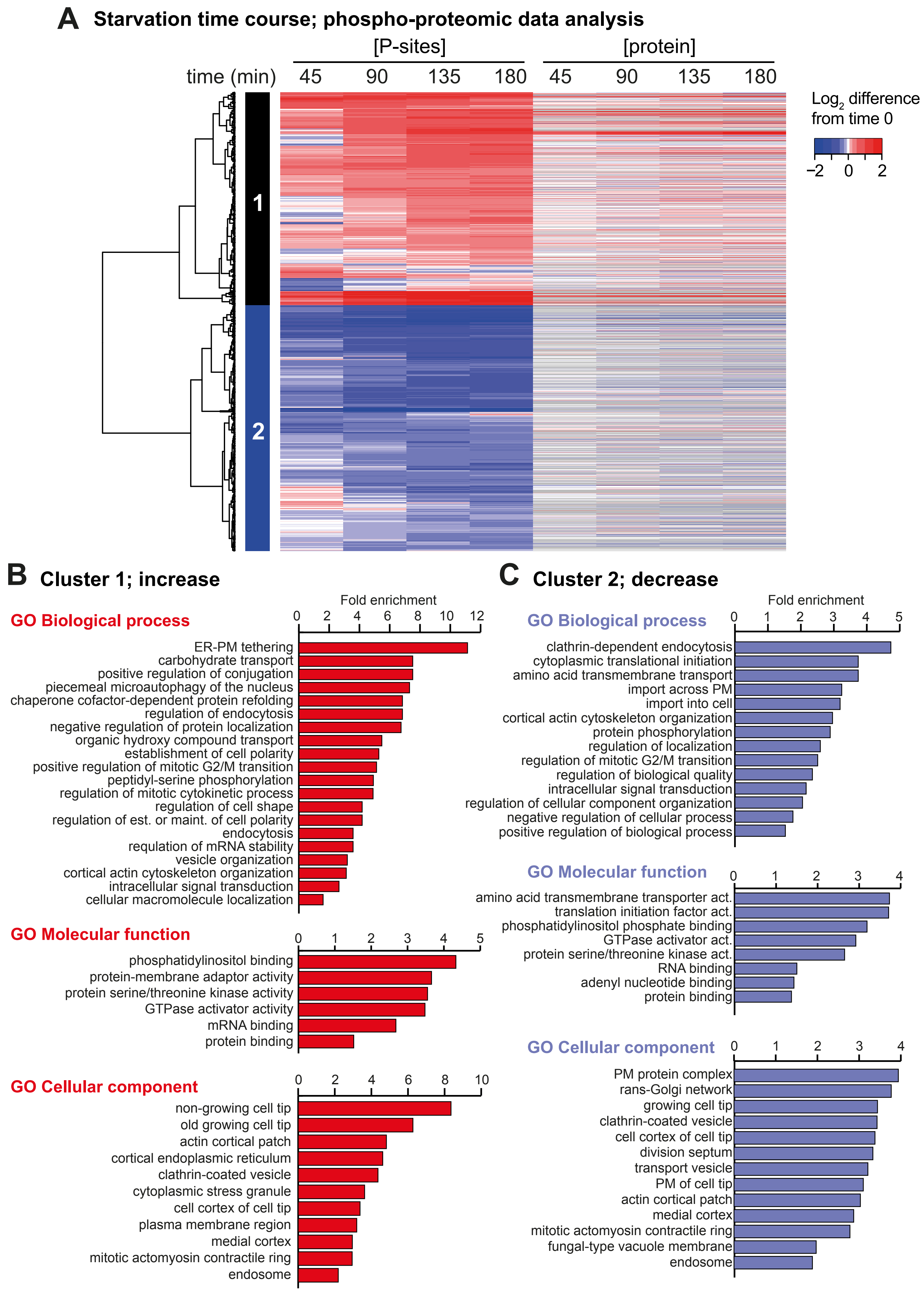

Supplement: S4 Fig — Changes in the phosphoproteome of heterothallic cells in a time course of nitrogen starvation starting at t0 = plating of cells on MSL-N plates 2 h after transfer to liquid MSL-N. (A) Heatmap of the significant changes in 3,100 phosphosites during nitrogen starvation, showing 2 major clusters of sites whose phosphorylation increase (1) or decrease (2). Corresponding protein dynamics is shown on the right with missing values in gray. Regression analysis estimated that about 78% of phosphosite level changes are independent of the changes in protein levels (see Fig 3C). The underlying data can be found in S1 Table. (B) Significant fold enrichment in GO annotations for biological processes, molecular functions, and cellular components of proteins containing one or several sites showing phosphorylation increase during nitrogen starvation. (C) Significant fold enrichment in GO annotations for biological processes, molecular functions, and cellular components of proteins containing one or several sites showing phosphorylation decrease during nitrogen starvation. Significance levels were assessed by Fisher’s exact test and corrected for false discovery rate. (PDF) [file pbio.3002963.s004.pdf]
