# Supplementary material for: Proteomic and phosphoproteomic analyses reveal that TORC1 is reactivated by pheromone signaling during sexual reproduction in fission yeast
Source: PLoS Biol. 2024 Dec 20;22(12):e3002963. doi: 10.1371/journal.pbio.3002963 (PMC11750111; doi:10.1371/journal.pbio.3002963)

Bérard, Figure S5

Mating time course; proteomic data analysis

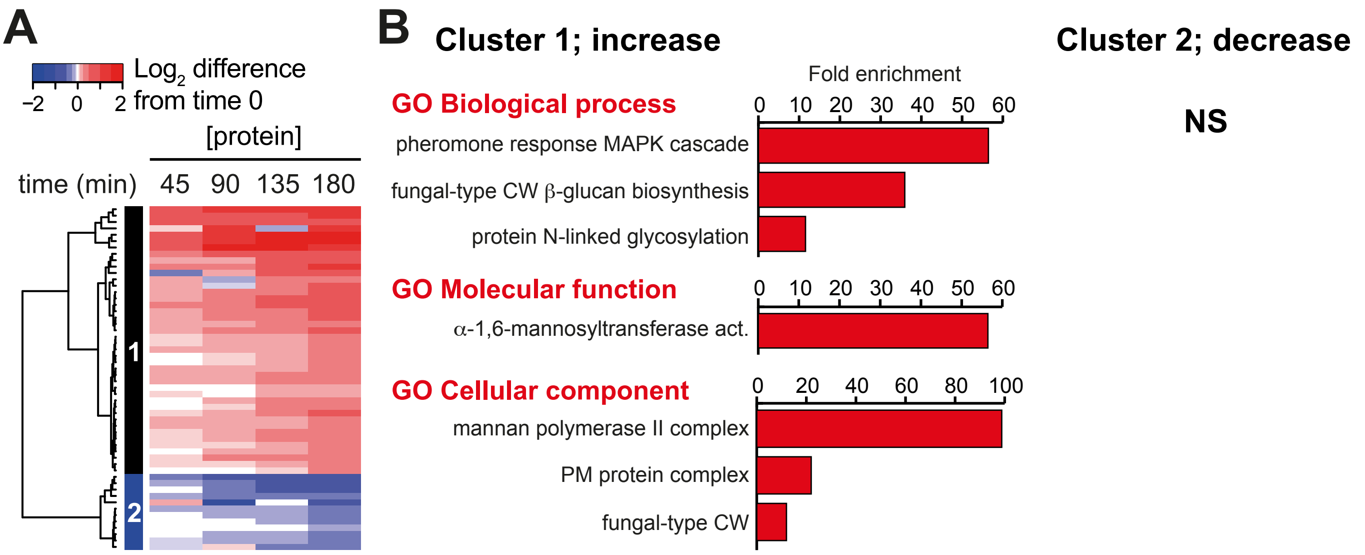

Mating time course; phospho-proteomic data analysis

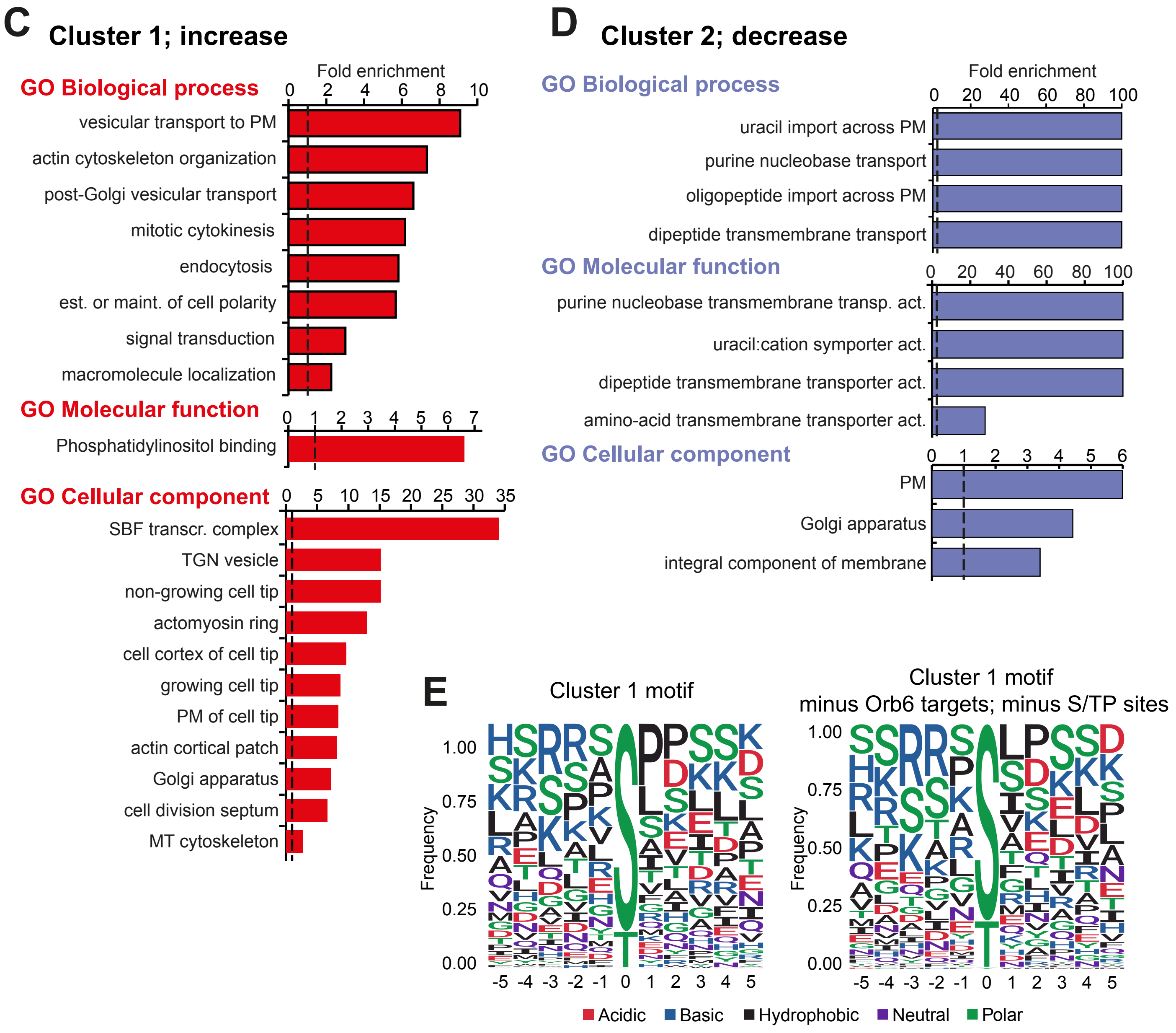

Supplement: S5 Fig — Changes in the proteome and phosphoproteome of mating cells in a time course starting at t0 = cell mixing on MSL-N plates in the dark. The cells were pre-grown separately in liquid MSL-N for 2 h. All Data are corrected for changes happening during starvation in absence of a mating partner. (A) Heatmap of the significant changes in the levels of 54 proteins during mating, showing 2 major clusters of proteins whose level increase (1) or decrease (2). The underlying data can be found in S1 Table. (B) Significant fold enrichment in GO annotations for biological processes, molecular functions, and cellular components in proteins whose level increases during mating. No significant enrichment was found for the few proteins whose levels decrease. (C) Significant fold enrichment in GO annotations for biological processes, molecular functions, and cellular components of proteins containing one or several sites showing phosphorylation increase during nitrogen starvation. (D) Significant fold enrichment in GO annotations for biological processes, molecular functions, and cellular components of proteins containing one or several sites showing phosphorylation decrease during nitrogen starvation. Significance levels were assessed by Fisher’s exact test and corrected for false discovery rate. (E) Analysis of the protein sequence surrounding serine and threonine residues phosphorylated during mating. The height of the amino acid one-letter code represents the frequency. The logo on the left was made using all 192 increasing phosphosites. For the one on the left, all proline-directed sites and Orb6 substrates were removed, leaving 122 phosphosites. (PDF) [file pbio.3002963.s005.pdf]
