# Supplementary material for: Proteomic and phosphoproteomic analyses reveal that TORC1 is reactivated by pheromone signaling during sexual reproduction in fission yeast
Source: PLoS Biol. 2024 Dec 20;22(12):e3002963. doi: 10.1371/journal.pbio.3002963 (PMC11750111; doi:10.1371/journal.pbio.3002963)

Bérard, Figure S6

Fusion time course; proteomic data analysis

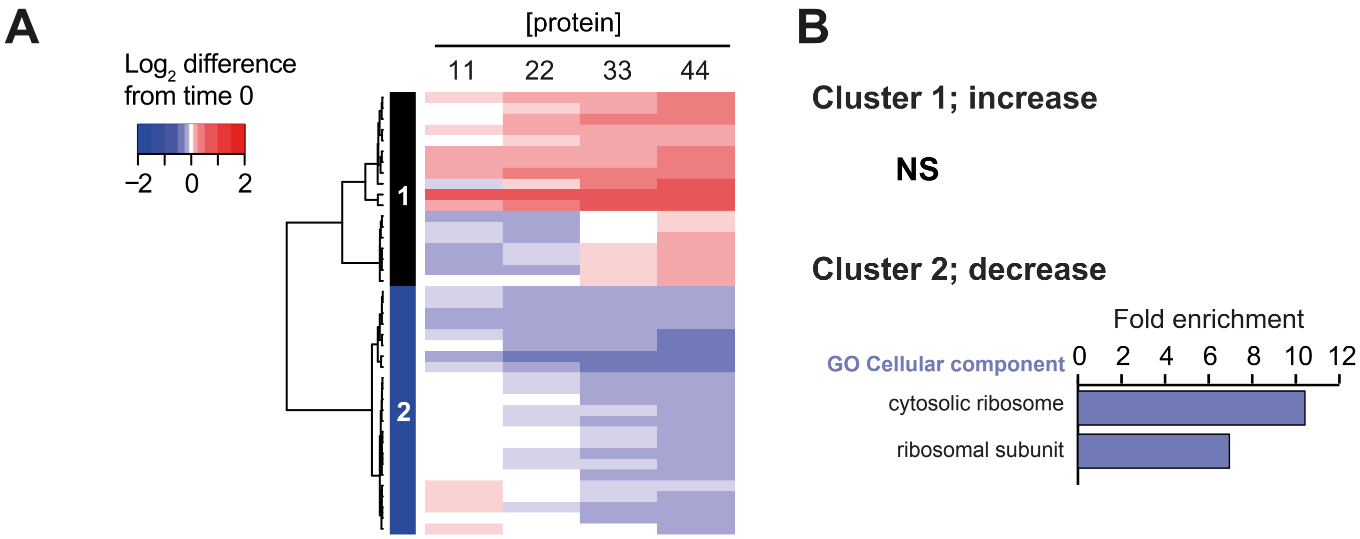

Fusion time course; phospho-proteomic data analysis

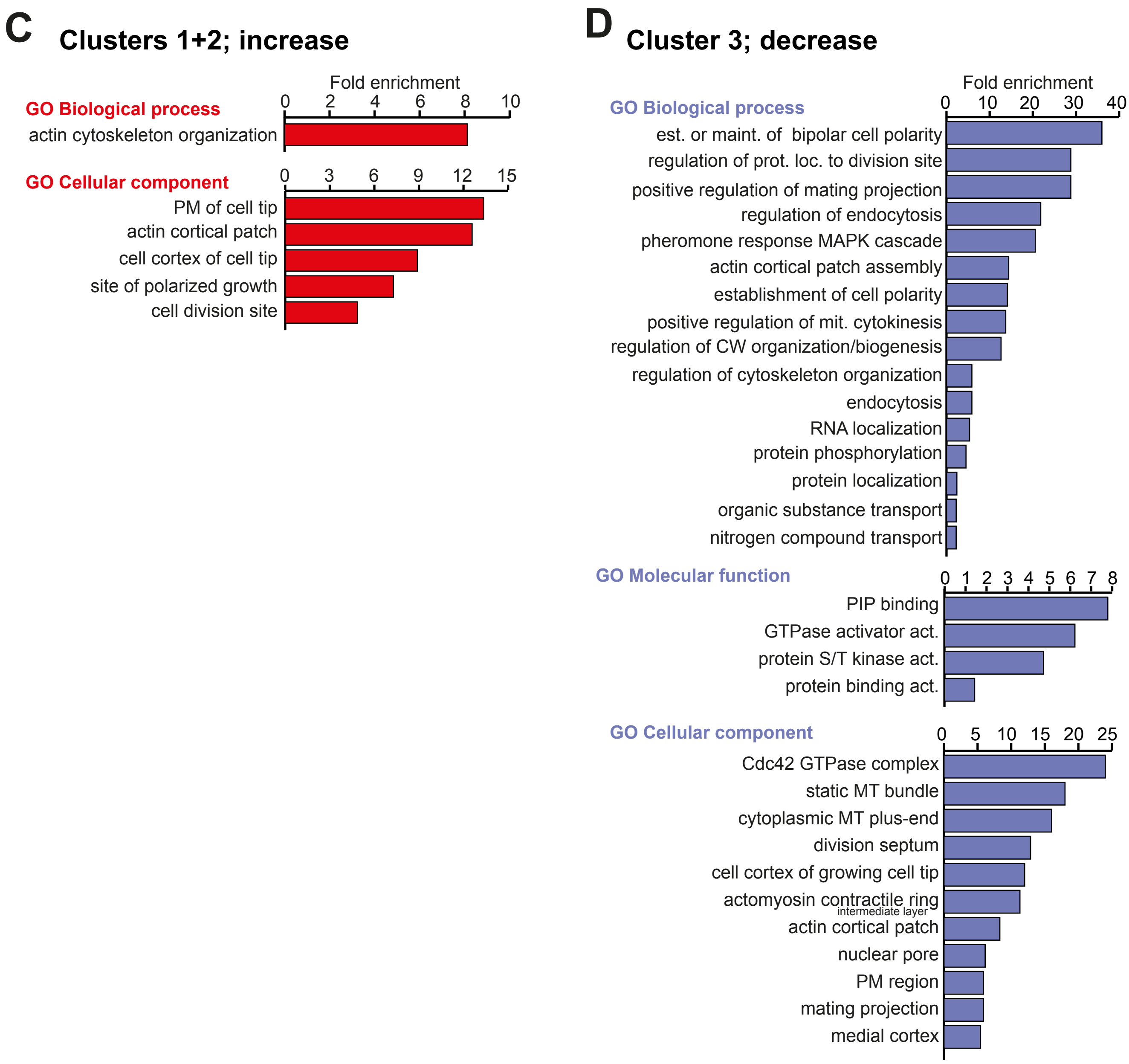

Supplement: S6 Fig — Changes in the proteome and phosphoproteome in a time course starting at t0 = light exposure. h- and h+ cycΔ5 fus1opto cells were pre-grown separately in liquid MSL-N for 2 h, mixed on plates for 150 in the dark before illumination. (A) Heatmap of the significant changes in the levels of 41 proteins during mating, showing 2 major clusters of proteins whose level increase (1) or decrease (2). Note that cluster 1 can be separated in 2 subclusters with levels increase at 11 or 33 min. The underlying data can be found in S1 Table. (B) Significant fold enrichment in GO annotations for cellular components of proteins whose level decreases during mating. No significant enrichment was found for the few proteins whose levels increase. (C) Significant fold enrichment in GO annotations for biological processes and cellular components of proteins containing one or several sites showing phosphorylation increase during cell–cell fusion. (D) Significant fold enrichment in GO annotations for biological processes, molecular functions, and cellular components of proteins containing one or several sites showing phosphorylation decrease during nitrogen starvation. Significance levels were assessed by Fisher’s exact test and corrected for false discovery rate. (PDF) [file pbio.3002963.s006.pdf]
