# Supplementary material for: Proteomic and phosphoproteomic analyses reveal that TORC1 is reactivated by pheromone signaling during sexual reproduction in fission yeast
Source: PLoS Biol. 2024 Dec 20;22(12):e3002963. doi: 10.1371/journal.pbio.3002963 (PMC11750111; doi:10.1371/journal.pbio.3002963)
Supplement: S3 Table — (PDF) [file pbio.3002963.s010.pdf]

Table S3: Strains used in this study

STRAINS	GENOTYPE	IN FIGURE	SOURCE
**YSM4130**	*h90 fus1N(1-793)-CIBN:kanMX ura4+:pfus1:Cry2PHR-fus1C(796-1372)-sfGFP myo52-tdtomato:natMX lys3:pmap3:mtagBFP2:BleMX. ade6+ leu1+*	1B,1C	This study
**YSM1396**	*h90 ad6+ leu1+ ura4+*	1D,2A,5A-D, 7, S6A,S1A,S1B	Lab stock
**YSM4131**	*h90 myo52-tdtomato:natMX fus1Δ::hphMX ade6+ leu1+ ura4-D18*	1D	This study
**YSM4132**	*h90 fus1N(1-793)-CIBN:kanMX myo52-tdtomato:natMX ade6+ leu1+ ura4-D18*	1D	This study
**YSM4133**	*h90 ura4+:pfus1:Cry2PHR-fus1C(796-1372)-sfGFP fus1Δ::hphMX myo52-tdtomato:natMX ade6+ leu1+*	1D	This study
**YSM4134**	*h90 fus1N(1-793)-CIBN:kanMX ura4+:pfus1:Cry2PHR-fus1C(796-1372)-sfGFP myo52-tdtomato:natMX ade6+ leu1+*	1D,1E,S1C	This study
**AV2038**	*h- cig1Δ::bsdMX cig2Δ::natMX puc1Δ::bleMX crs1Δ::kanMX rem1Δ::kanMX ade6+ leu1+ ura4+*	2B,2C,S1A,S1B	*[32]*
**AV2050**	*h+ cig1Δ::bsdMX cig2Δ::natMX puc1Δ::bleMX crs1Δ::kanMX rem1Δ::kanMX ade6+ leu1+ ura4+*	2B,2C,S1A,S1B	*[32]*
**AV2343**	*h- ad6+ leu1+ ura4+*	2B,5A,5B,5C,7B,S6A,S1A,S1B	*[32]*
**AV2344**	*h+ ad6+ leu1+ ura4+*	2B,S1A,S1B	*[32]*
**YSM4135**	*h- cig1Δ::bsdMX cig2Δ::natMX puc1Δ::bleMX crs1Δ::kanMX rem1Δ::kanMX fus1N(1-793)-CIBN:hphMX ura4+:pfus1:Cry2PHR- fus1C(796-1372)-sfGFP ade6+ leu1+*	2C,3B,3C,3D,3E,3F,3G,3H,3I,4B,4C,4D,4E,4F,4G,4H,5A,S1,S2,S3,S4,S5	This study
**YSM4136**	*h+ cig1Δ::bsdMX cig2Δ::natMX puc1Δ::bleMX crs1Δ::kanMX rem1Δ::kanMX fus1N(1-793)-CIBN:hphMX ura4+:pfus1:Cry2PHR- fus1C(796-1372)-sfGFP ade6+ leu1+*	3B,3C,3D,3E,3F,3G,3H,3I,4B,4C,4D,4E,4F,4G,4H,5A,S2,S3,S4,S5	This study
**YSM4137**	*h+ cig1Δ::bsdMX cig2Δ::natMX puc1Δ::bleMX crs1Δ::kanMX rem1Δ::kanMX fus1N(1-793)-CIBN:hphMX ura4+:pfus1:Cry2PHR- fus1C(796-1372)-sfGFP ade6+:pact1:mcherry:tdh1term:patMX leu1+*	2C,S1C	This study
**YSM4138**	*h90 rps601Δ::hphMX rps602(SS235236AA):kanMX*	5A,5D,7A	This study
**YSM4139**	*h+ rps601Δ::hphMX rps602(SS235236AA):kanMX*	5A	This study
**YSM4063**	*h- tor2(S1837E):kanMX ade6+ leu1+ ura4+*	5C	[117]
**YSM4140**	*h90 tor2(S1837E):kanMX ade6+ leu1+ ura4+*	5C,7E,7F	This study
**YSM1371**	*h+ ade6+ leu1+ ura4+*	5D	*Lab stock*
**YSM4141**	*h90 psk1Δ::natMX ade6+ leu1+ ura4+*	5D	This study
**YSM4142**	*h90 psk1Δ::natMX ade6+ leu1+ ura4+*	5D,7A	This study
**YSM4143**	*h- sxa2Δ::hphMX ade6+ leu1+ ura4+*	5E	This study
**YSM4144**	*h90 tor2-ts10 ade6+ leu1+ ura4+*	7F,7G	This study
**YSM4145**	*h90 rps601Δ::hphMX ade6+ leu1+ ura4+*	7A	This study
**YSM4146**	*h90 ade6+ leu1-32 ura4+*	7A,7B	Lab stock
**YSM4147**	*h90 ade6+ leu1-32 ura4*	7A	This study
**YSM4148**	*h90 psk1Δ::natMX ade6+ leu1-32 ura4*	7A	This study
**YSM4149**	*h90 rps602Δ::kanMX ade6+ leu1-32 ura4+*	7A	This study
**YSM4150**	*h90 rps602Δ::kanMX ade6+ leu1-32 ura4+*	7A	This study
**YSM4151**	*h90 rps601Δ::hphMX ade6+ leu1-32 ura4+*	7A	This study
**YSM4152**	*h90 rps601Δ::hphMX ade6+ leu1-32 ura4+*	7A	This study
**YSM4153**	*h- ade6+ leu1-32 ura4+*	7B	Lab stock
**YSM4154**	*h90 ade6+ leu1-32 ura4+*	7C	Lab stock
**YSM4155**	*h90 tco89Δ::hphMX ade6+ leu1+ ura4+*	7H	This study
**YSM4156**	*h- CFP-atg8:leu1+ sxa2Δ::natMX ade6+ leu1-32 ura4+*	6A,6B,6C,6D,6E,6F,S7B	This study
**YSM4157**	*h- atg1Δ::kanMX CFP-atg8:leu1+ sxa2Δ::natMX ade6+ leu1-32 ura4+*	6A,6B,6C,6D,6F,S7B	This study
**YSM4158**	*h- atg1Δ::kanMX CFP-atg8:leu1+ ade6+ leu1-32 ura4+*	6D	*[85]*
**YSM4170**	*h- atg5Δ::kanMX CFP-atg8:leu1+ ade6+ leu1-32 ura4+*	6D	*[85]*
**YSM4171**	*h- atg7Δ::kanMX CFP-atg8:leu1+ ade6+ leu1-32 ura4+*	6D	*[85]*

STRAINS	GENOTYPE	IN FIGURE	SOURCE
**YSM4172**	*h- atg12Δ::kanMX CFP-atg8:leu1+ ade6+ leu1-32 ura4+*	6D	*[85]*
**YSM4173**	*h- atg18aΔ::kanMX CFP-atg8:leu1+ ade6+ leu1-32 ura4+*	6D	*[85]*
**YSM4174**	*h- atg5Δ::kanMX CFP-atg8:leu1+ sxa2Δ::natMX ade6+ leu1-32 ura4+*	6D,6E,6F	This study
**YSM4175**	*h- atg18aΔ::kanMX CFP-atg8:leu1+ sxa2Δ::natMX ade6+ leu1-32 ura4+*	6D,S7C	This study
