# Supplementary material for: Proteomic and phosphoproteomic analyses reveal that TORC1 is reactivated by pheromone signaling during sexual reproduction in fission yeast
Source: PLoS Biol. 2024 Dec 20;22(12):e3002963. doi: 10.1371/journal.pbio.3002963 (PMC11750111; doi:10.1371/journal.pbio.3002963)
Supplement: S4 Table — (PDF) [file pbio.3002963.s011.pdf]

Table S4: Plasmids used in this study

PLASMID	DESCRIPTION	OBTAINED FROM	USAGE
**PSM693**	PFA6a-hphMX	Lab Stock	Template for PCR-based HR
**PSM2470**	3’region_fus1-5’region_fus1-fus1N$^{1-793}$-CIBN-3’UTR_fus1-term^{fus1}-kanMX-pFA6a	Infusion cloning	Truncation and tagging of Fus1N with CIBN at endogenous locus
**PSM2475**	pUra^{AfeI}-pfus1-cry2PHR-fus1C$^{796-1372}$-sfGFP-term^{nmt1}	Infusion cloning	Single integration of CRY2-Fus1C at *ura4*
**PAV0761**	pLys3^{BstZ17I}-pmap3-mTagBFP2-term^{ScAdh1}-bleMX	[116]	Single integration of pmap3-mTagBFP2 at *lys3*
**PSM3227**	3'region_fus1-5'region_fus1-fus1N$^{1-793}$-CIBN-3'UTR_fus1-term^{fus1}-hphMX-pFA6a	Infusion cloning	Endogenous integration
**PSM3295**	pAde6^{PmeI}-pact1-mcherry-term^{tdh1} -patMX	Infusion cloning	Single integration of pact1-mCherry at *ade6*
**PSM3571**	3'region_psk1-5'region_psk1-natMX-pFA6a	Infusion cloning	Deletion of *psk1*
**PSM3297**	3'region_rps602-5'region_rps602-kanMX-pFA6a	Infusion cloning	Deletion of *rps602*
**PSM3298**	3'region_rps601-5'region_rps601-hphMX-pFA6a	Infusion cloning	Deletion of *rps601*
**PSM3364**	pFA6-tco89-3'UTR-AfeI-5'UTR-hphMX	Infusion cloning	Deletion of *tco89*
**PSM3274**	3’UTRsxa2-5’UTRsxa2-pFA6a-natMX	Infusion cloning	Deletion of *sxa2*
