# Supplementary material for: Proteomic and phosphoproteomic analyses reveal that TORC1 is reactivated by pheromone signaling during sexual reproduction in fission yeast
Source: PLoS Biol. 2024 Dec 20;22(12):e3002963. doi: 10.1371/journal.pbio.3002963 (PMC11750111; doi:10.1371/journal.pbio.3002963)
Supplement: S1 Raw Images — For each figure panel, the panel is shown for reference. Each of the full-size, uncropped western blots is shown by itself and overlaid with the ladder. The relevant protein is indicated with an arrowhead. Asterisks mark lanes not included in the final figures. (PDF) [file pbio.3002963.s012.pdf]

Uncropped Western blots for the figures shown in:

Proteomic and phosphoproteomic analyses reveal that TORC1 is reactivated by pheromone signaling during sexual reproduction in fission yeast

Melvin Bérard[1,2], Laura Merlini[2] and Sophie G Martin[1,2,*]

For each figure panel, the panel is shown for reference. Each of the full-size, uncropped western blots is shown by itself and overlaid with the ladder. The relevant protein is indicated with an arrowhead. Asterisks mark lanes not included in the final figures.

Figure 5A

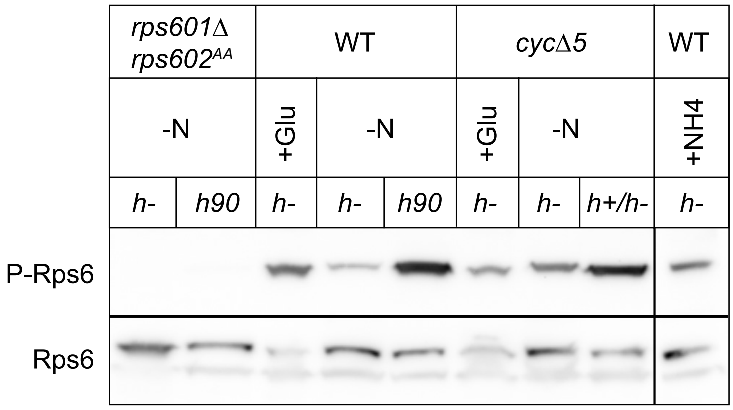

*Lane 9 not part of figure

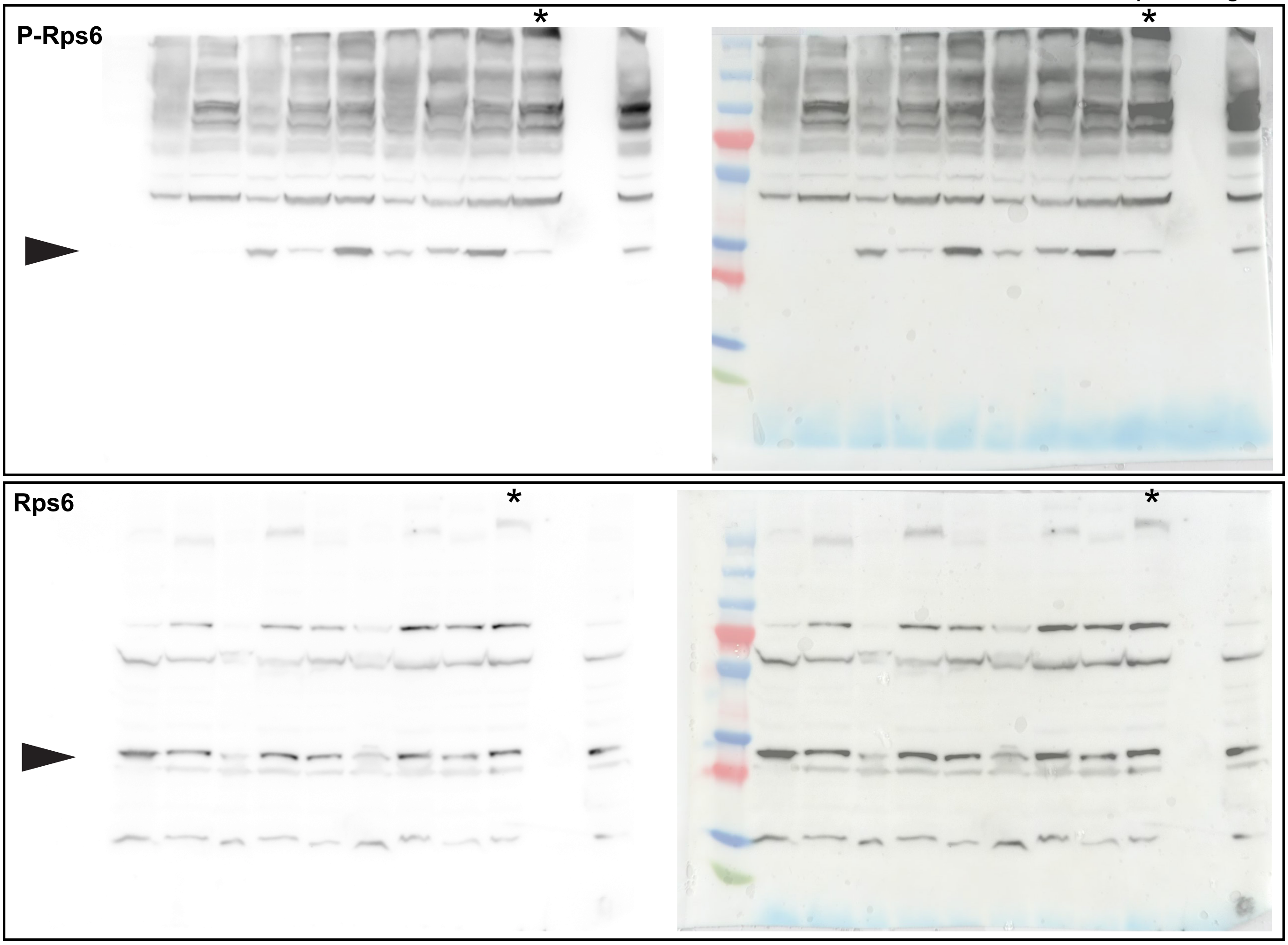

Figure 5B

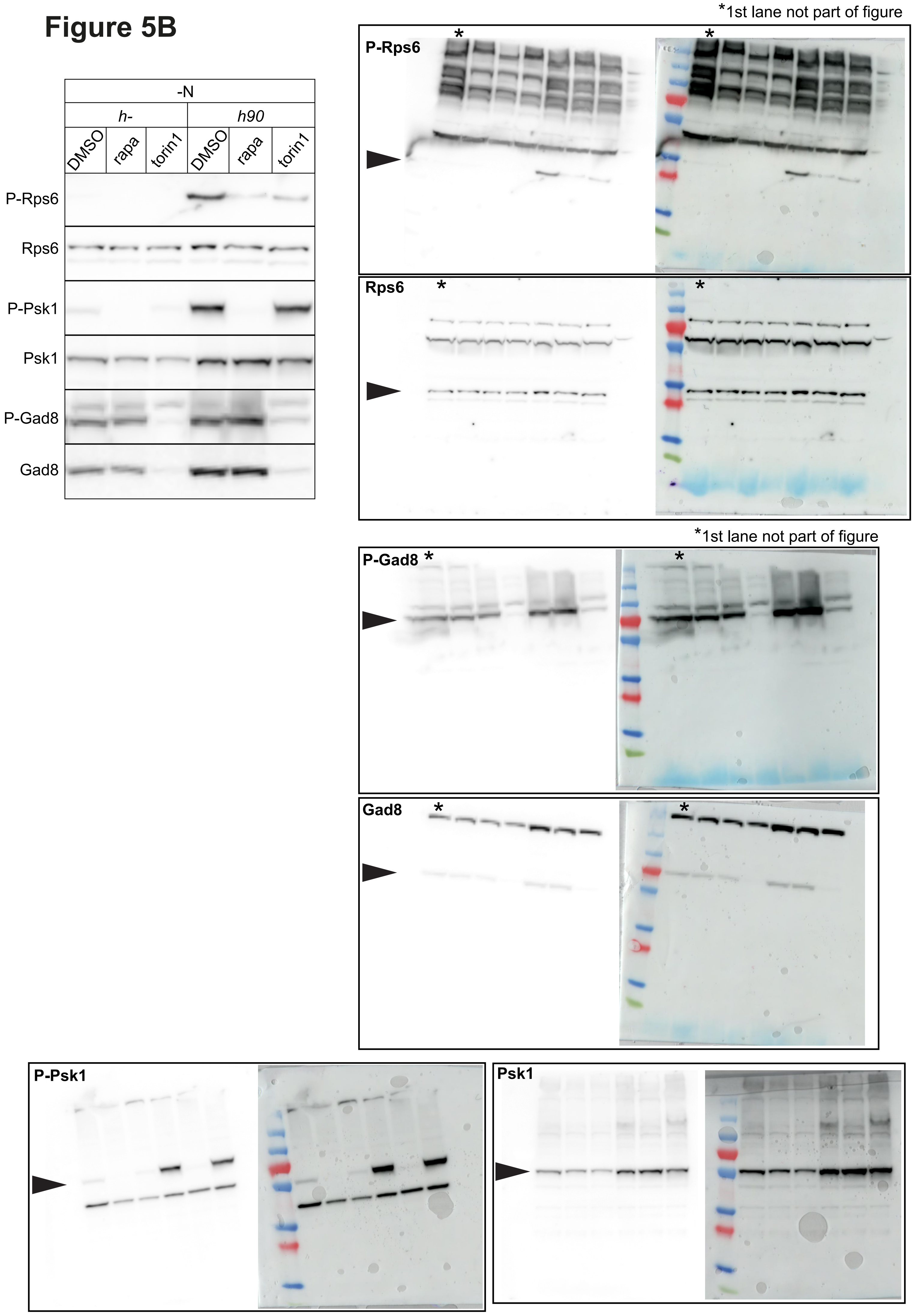
-N
h-
h90
DMSO
rapa
torin1
DMSO
rapa
torin1
P-Rps6
Rps6
P-Psk1
Psk1
P-Gad8
Gad8
*1st lane not part of figure
P-Rps6
Rps6
*1st lane not part of figure
P-Gad8
Gad8
P-Psk1
Psk1

Figure 5C

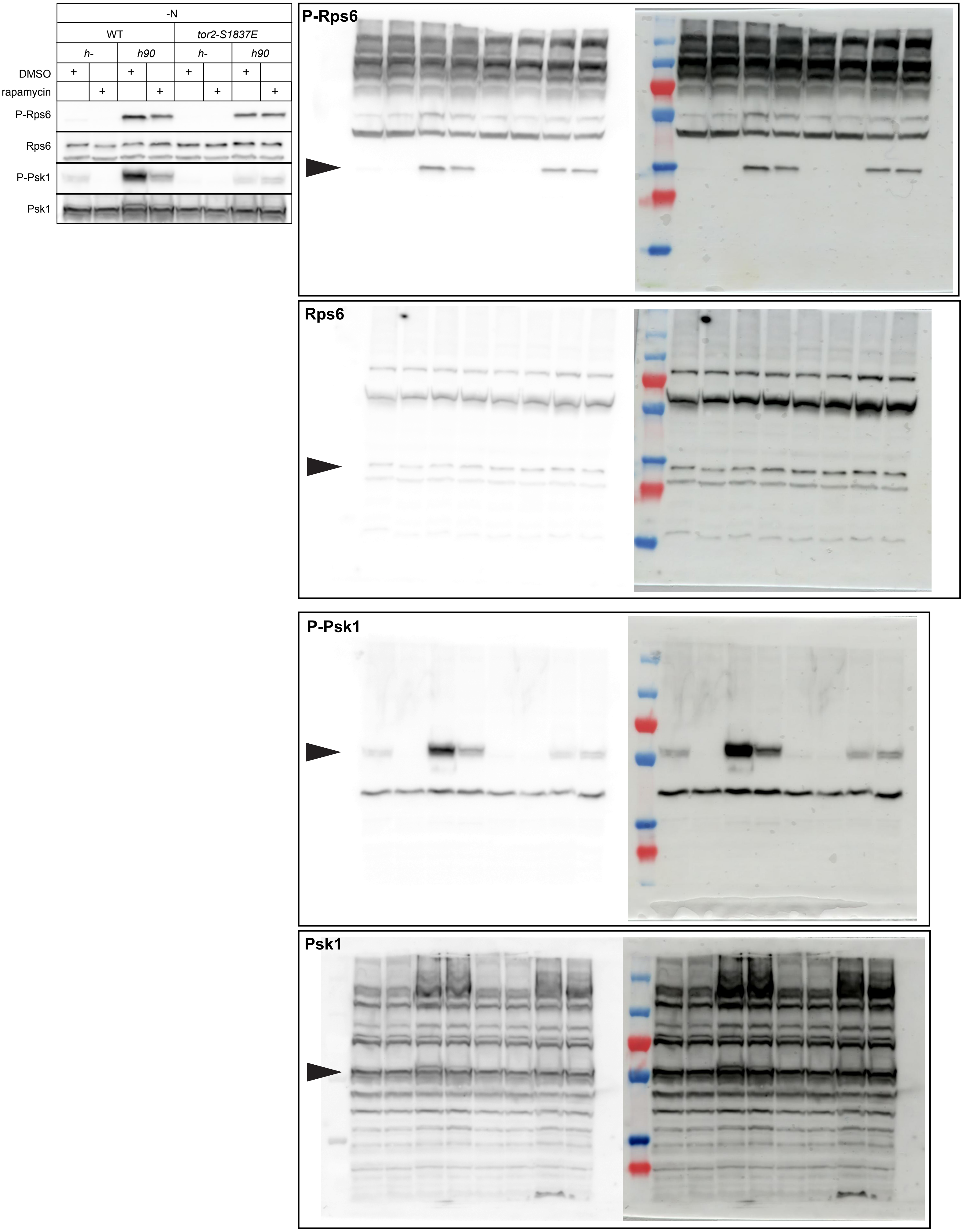
-N
WT
tor2-S1837E
h-
h90
h-
h90
DMSO
rapamycin
P-Rps6
Rps6
P-Psk1
Psk1
P-Rps6
Rps6
P-Psk1
Psk1

Figure 5D

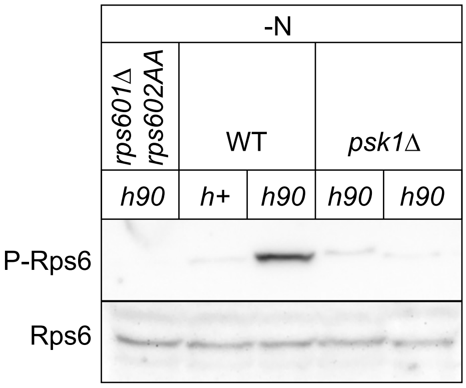

*Last two lanes not part of figure

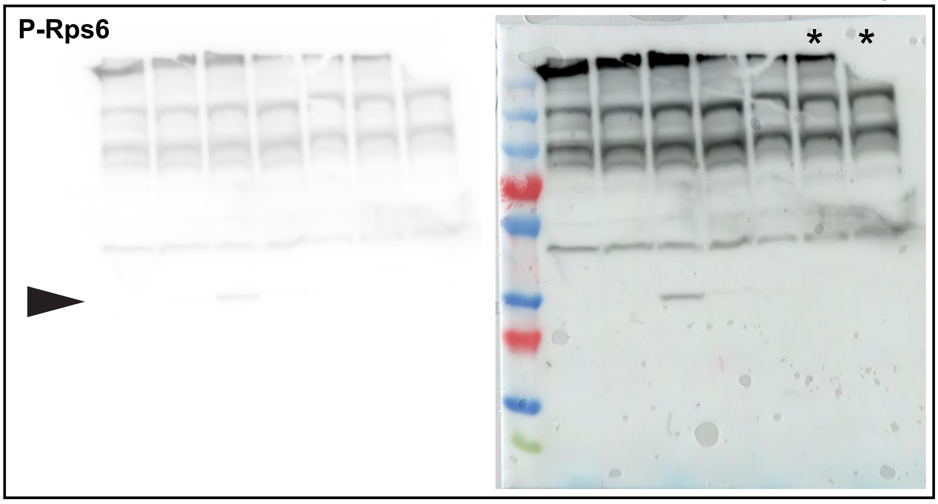

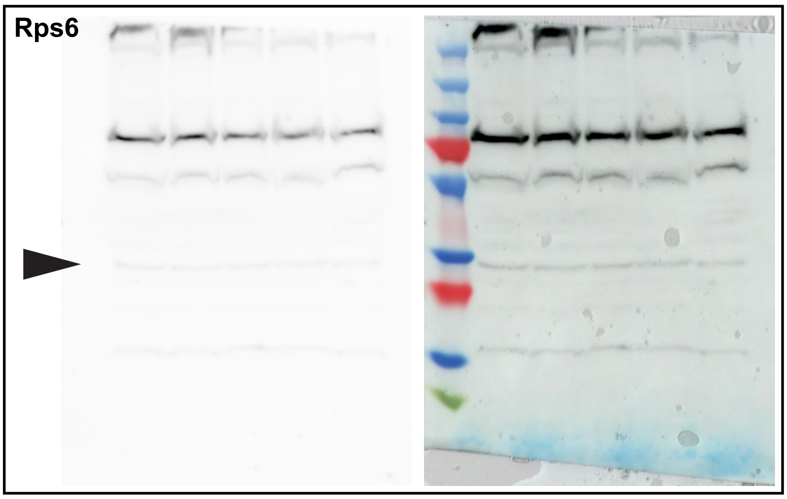

Figure 5E

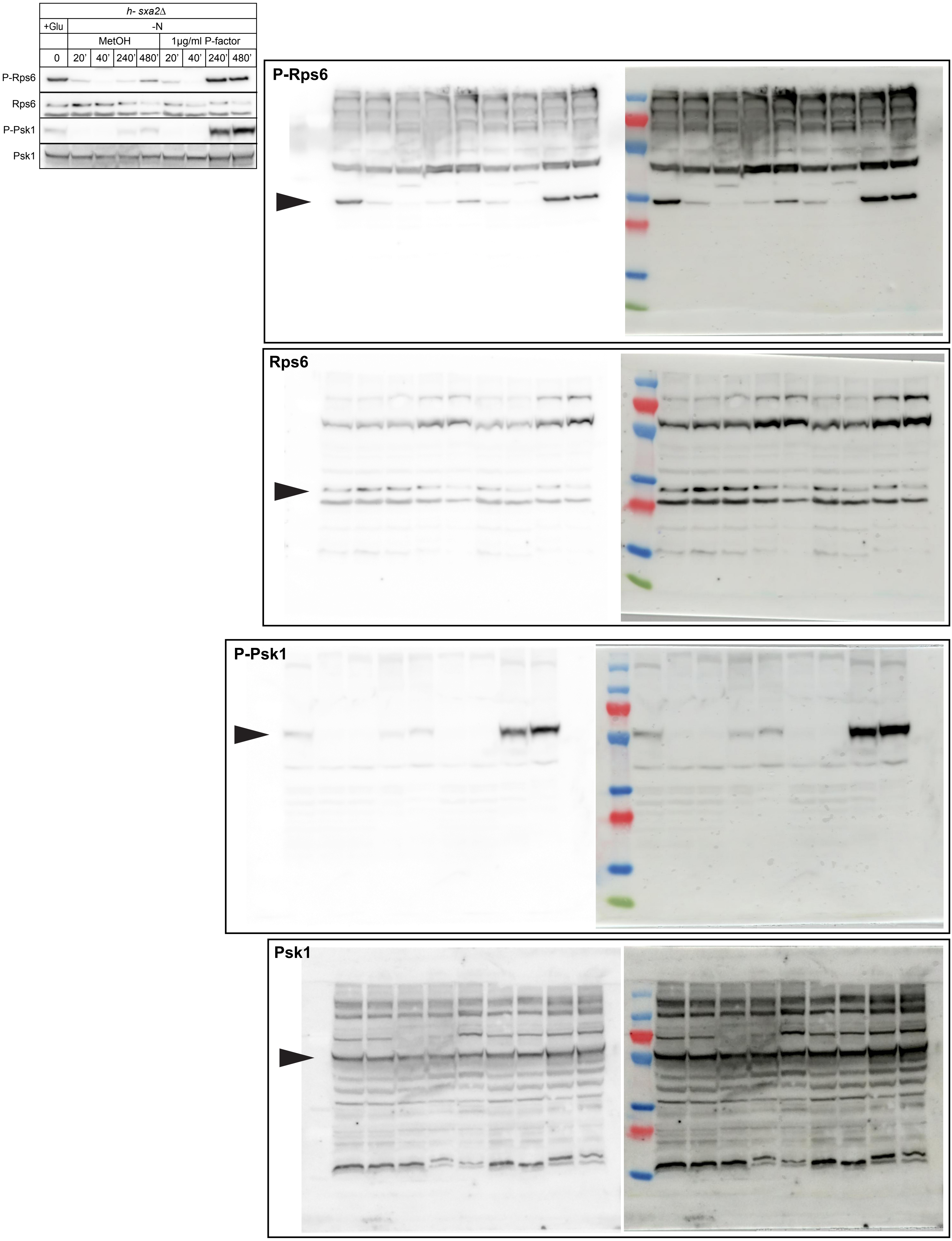
h- sxa2Δ
+Glu
-N
MetOH
1μg/ml P-factor
0
20'
40'
240'
480'
20'
40'
240'
480'
P-Rps6
Rps6
P-Psk1
Psk1
P-Rps6
Rps6
P-Psk1
Psk1

Figure 5F

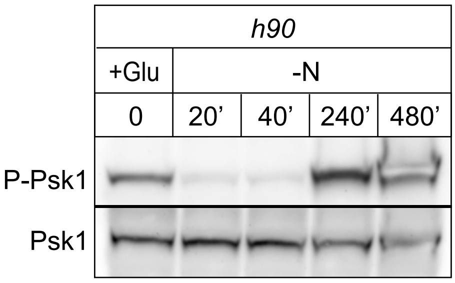

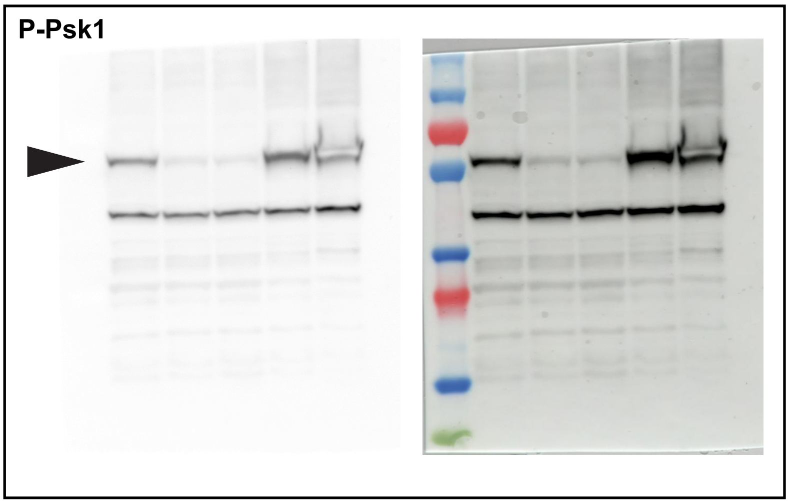

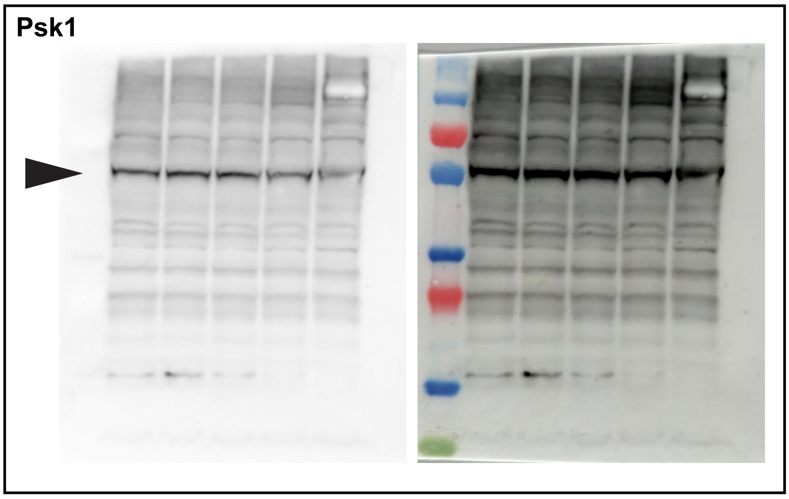

Figure 6B

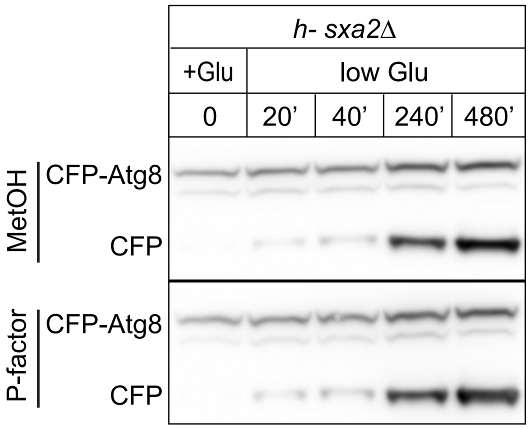
h- sxa2Δ
+Glu
low Glu
0
20’
40’
240’
480’
MetOH
CFP-Atg8
CFP
P-factor
CFP-Atg8
CFP

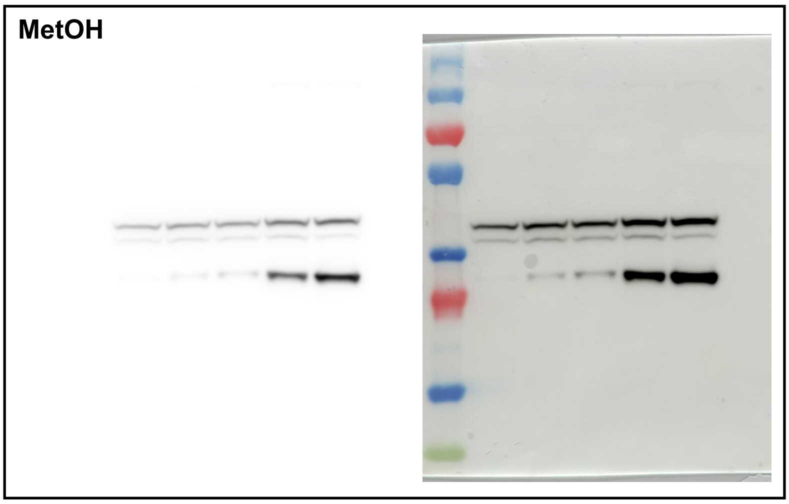
MetOH

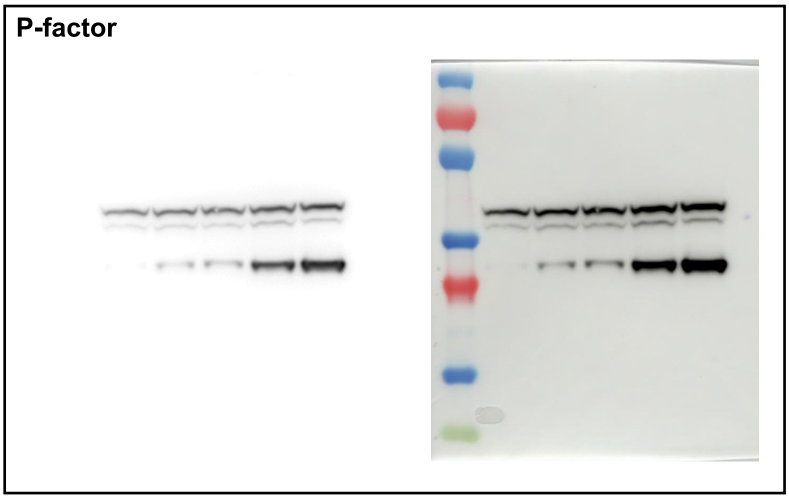
P-factor

Figure 6C

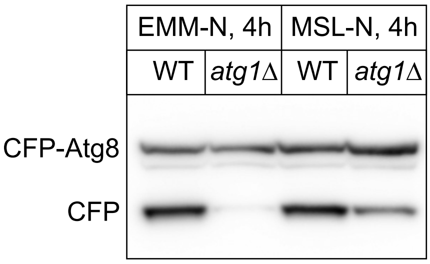

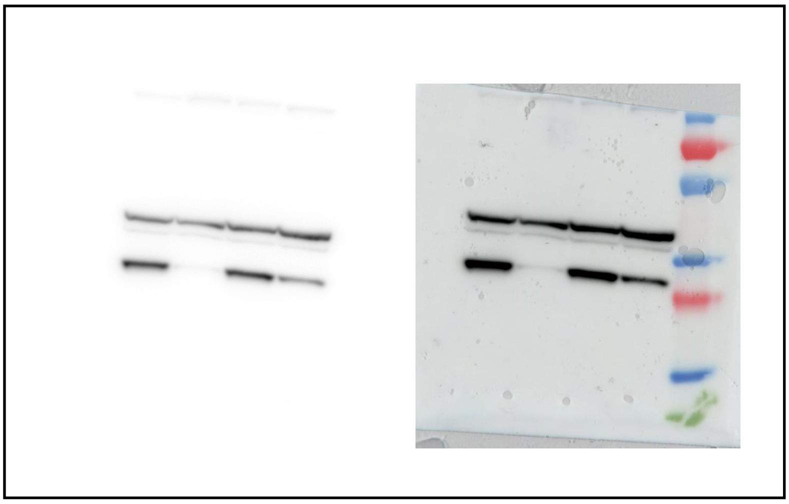

Figure 6D

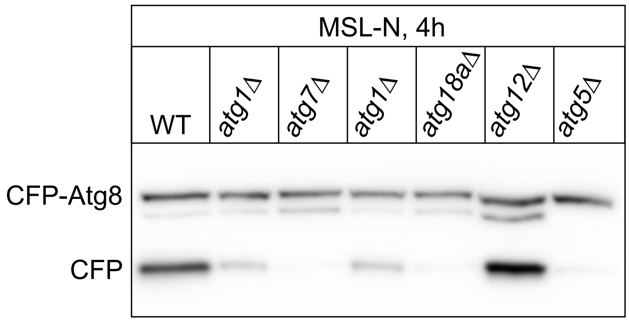

MSL-N, 4h
WT
atg1Δ
atg7Δ
atg1Δ
atg18aΔ
atg12Δ
atg5Δ
CFP-Atg8
CFP

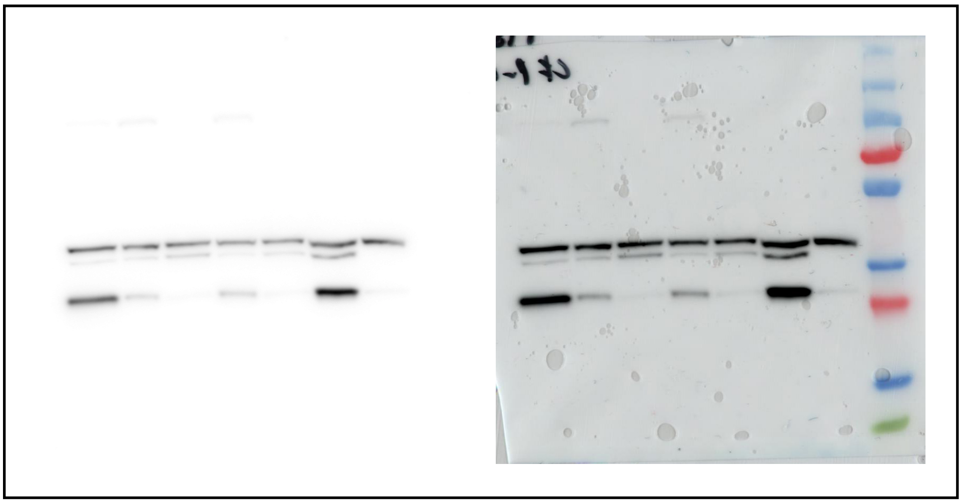

Figure 6F

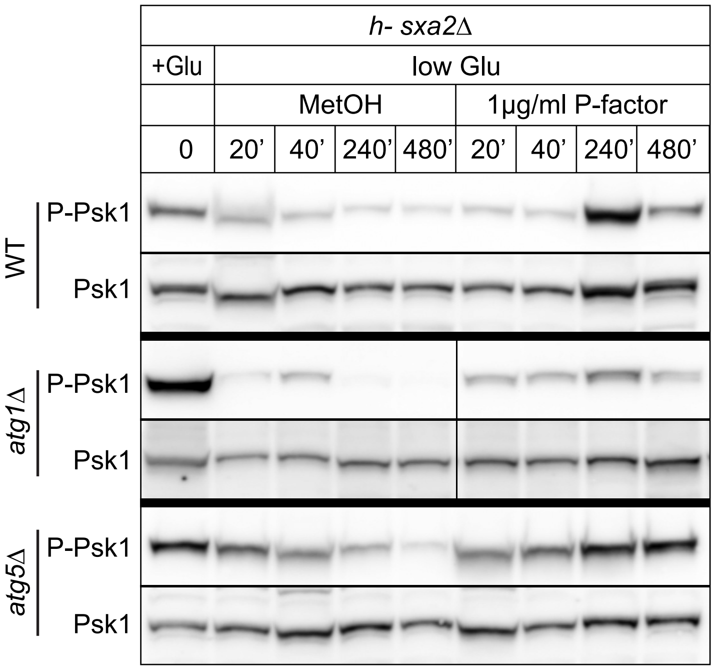

h- sxa2Δ
+Glu
low Glu
MetOH
1µg/ml P-factor
0
20'
40'
240'
480'
20'
40'
240'
480'
WT
P-Psk1
Psk1
atg1Δ
P-Psk1
Psk1
atg5Δ
P-Psk1
Psk1

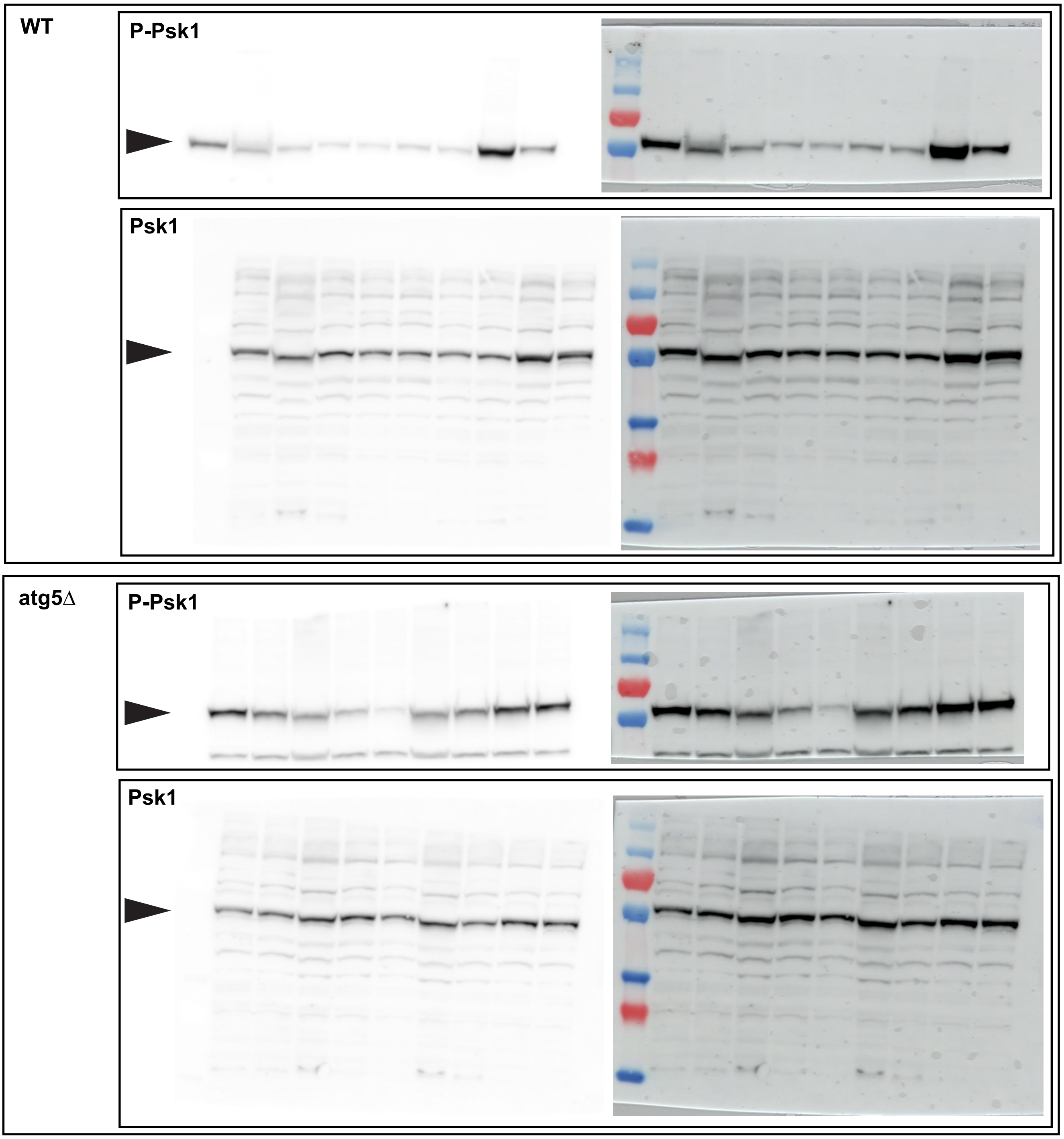

WT
P-Psk1
Psk1
atg5Δ
P-Psk1
Psk1

Figure 6F

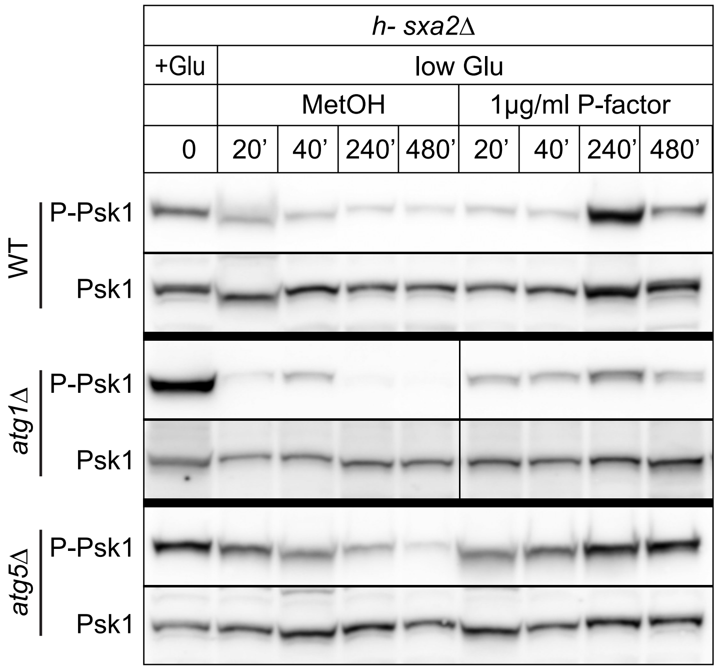

*Lanes 6 and 11 not part of the figure

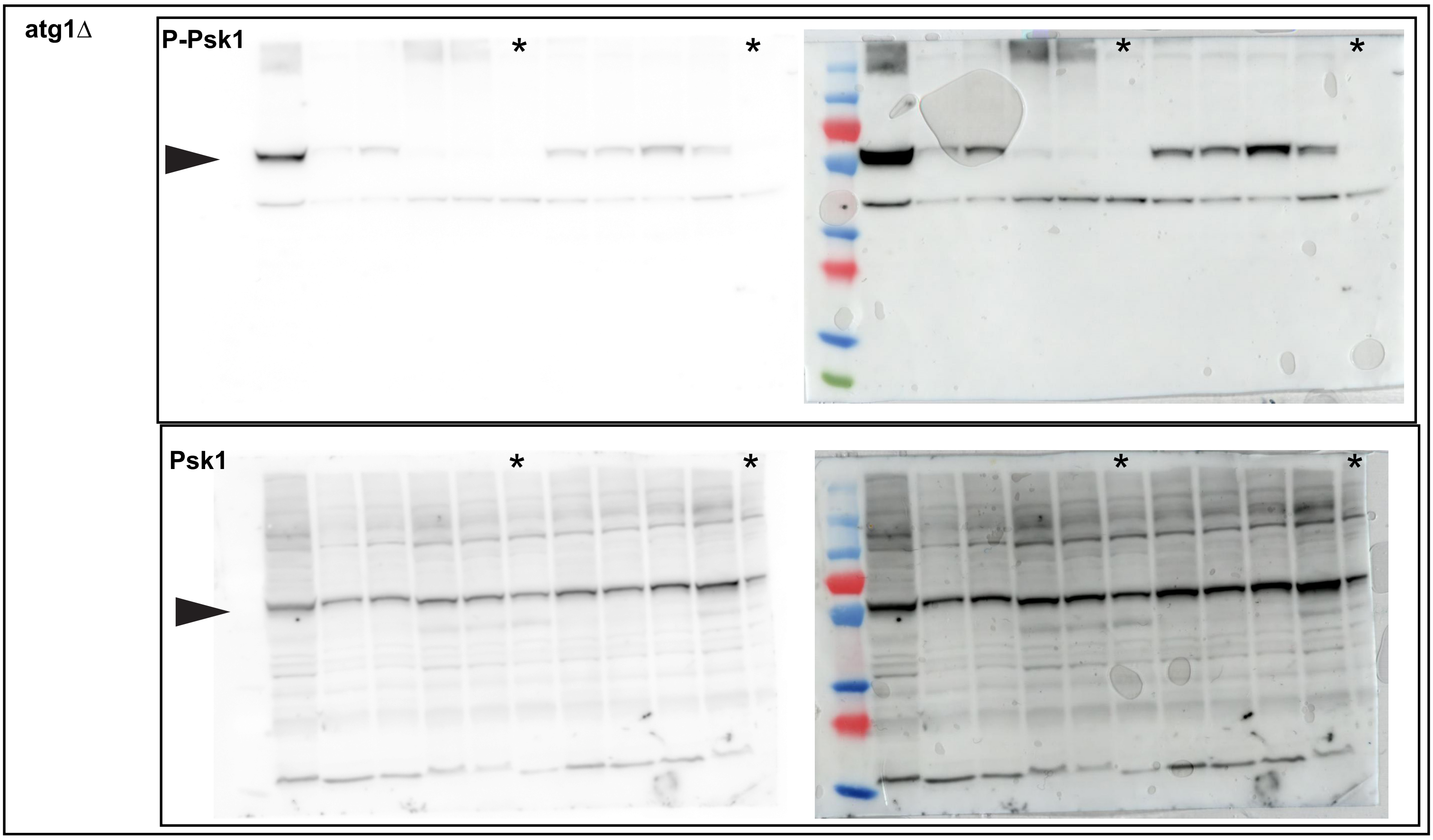

Figure 7B

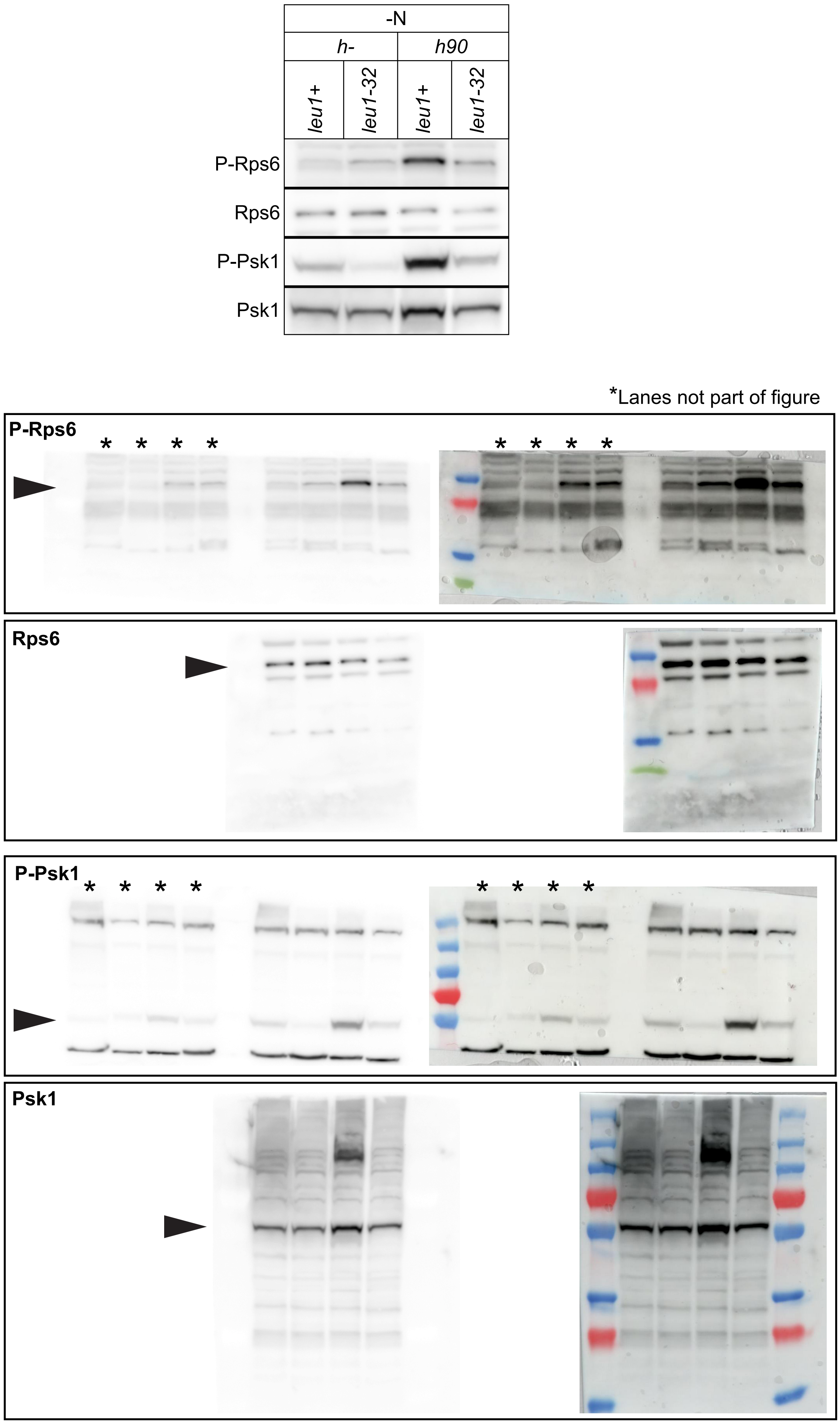
-N
h-
h90
leu1+
leu1-32
leu1+
leu1-32
P-Rps6
Rps6
P-Psk1
Psk1
*Lanes not part of figure
P-Rps6
Rps6
P-Psk1
Psk1

Figure S7A

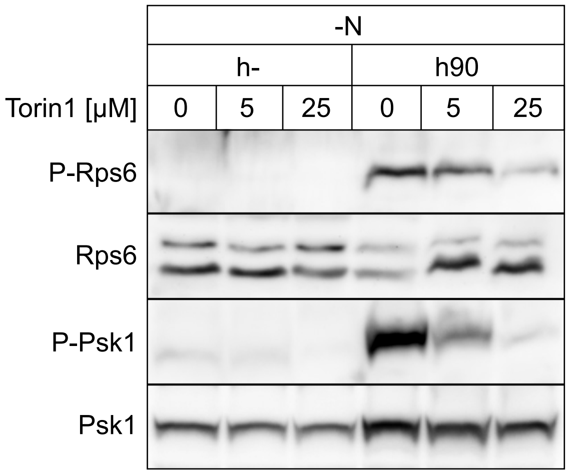
-N
h-
h90
Torin1 [μM]
0
5
25
0
5
25
P-Rps6
Rps6
P-Psk1
Psk1

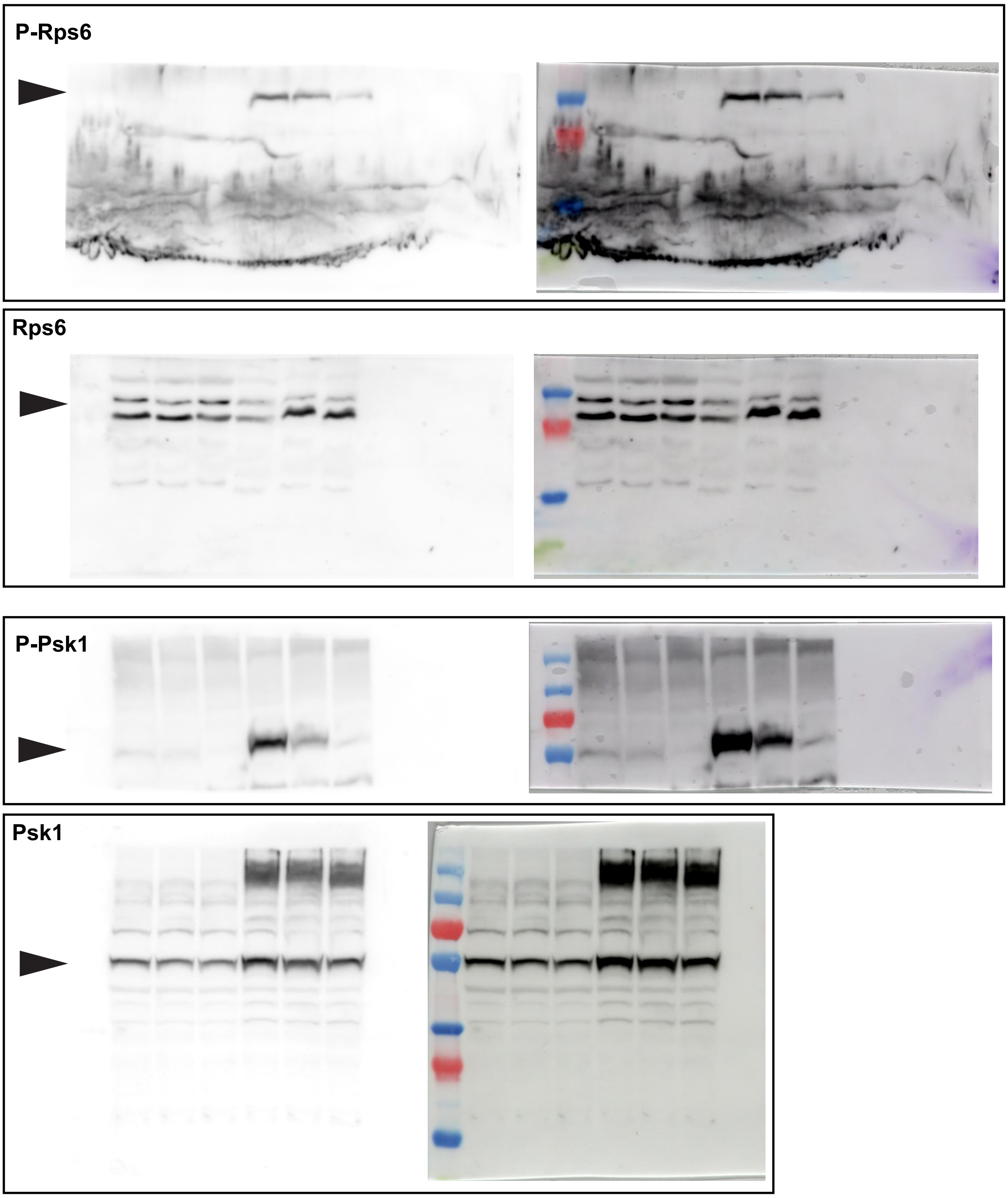
P-Rps6
Rps6
P-Psk1
Psk1

Figure S7B

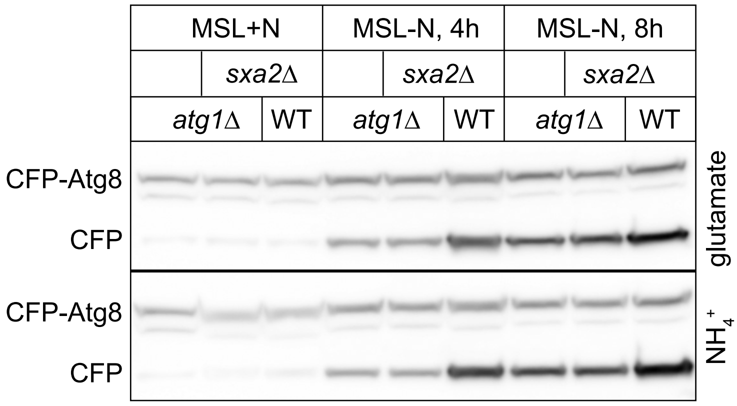
MSL+N
MSL-N, 4h
MSL-N, 8h
sxa2Δ
sxa2Δ
sxa2Δ
atg1Δ
WT
atg1Δ
WT
atg1Δ
WT
CFP-Atg8
CFP
CFP-Atg8
CFP
glutamate
NH4+

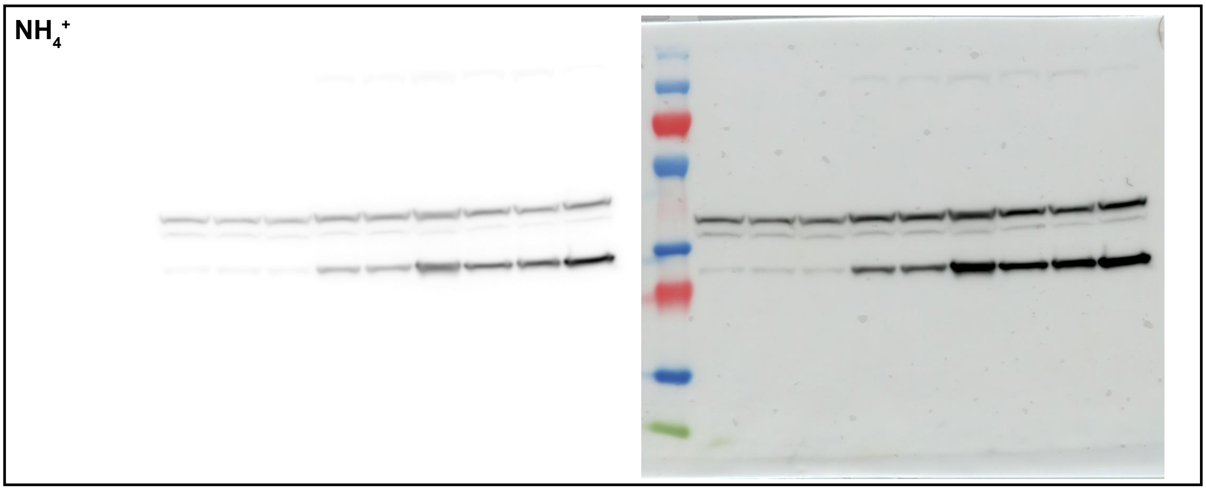
NH4+

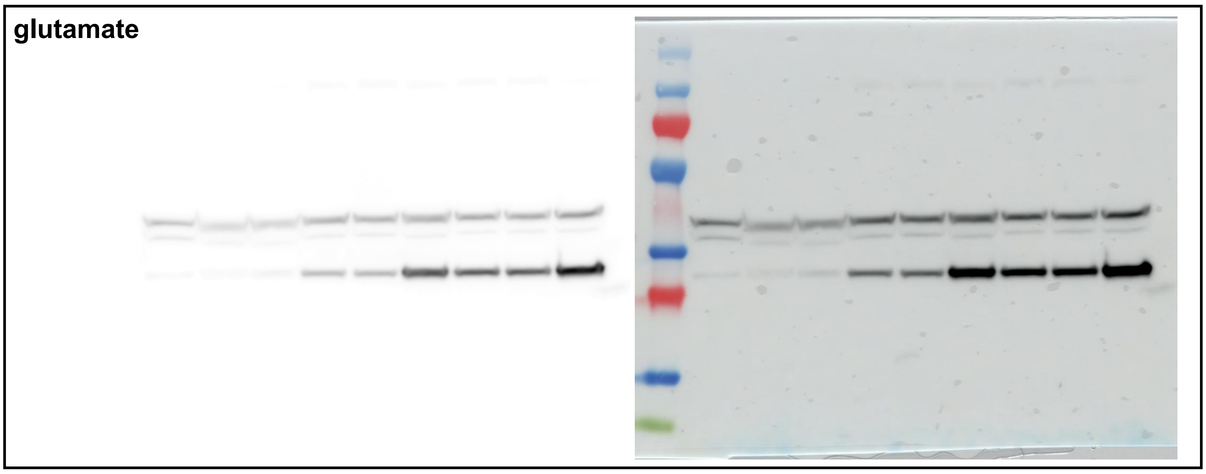
glutamate

Figure S7C

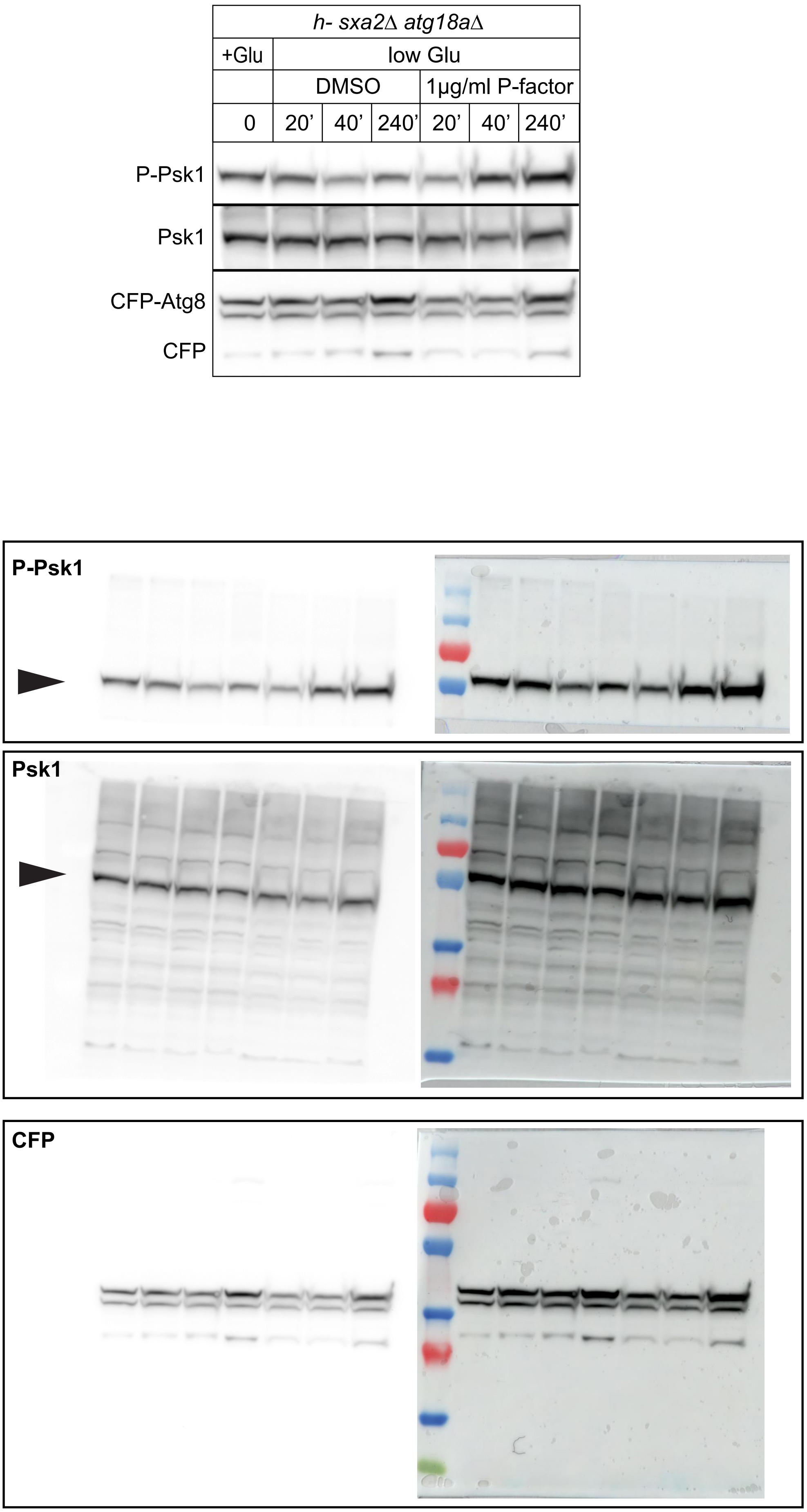
h- sxa2Δ atg18aΔ
+Glu
low Glu
DMSO
1µg/ml P-factor
0
20'
40'
240'
20'
40'
240'
P-Psk1
Psk1
CFP-Atg8
CFP
P-Psk1
Psk1
CFP

Figure S7D

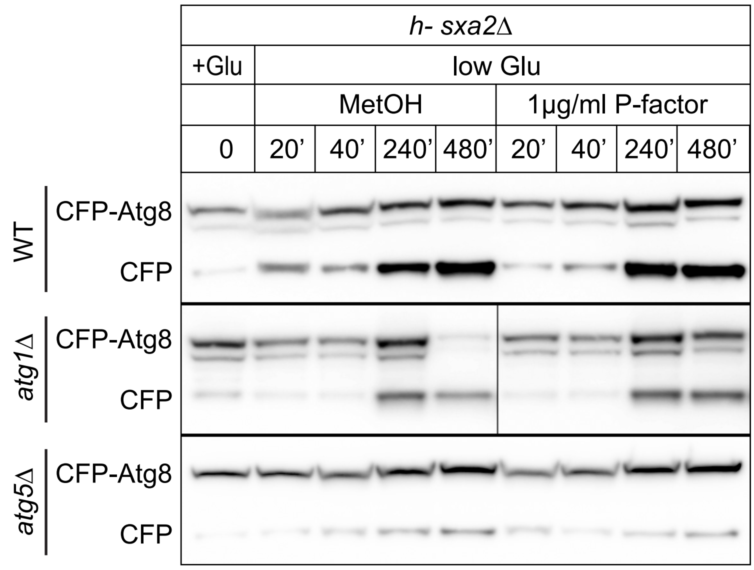

h- sxa2Δ
+Glu
low Glu
MetOH
1μg/ml P-factor
0
20’
40’
240’
480’
20’
40’
240’
480’
WT
CFP-Atg8
CFP
atg1Δ
CFP-Atg8
CFP
atg5Δ
CFP-Atg8
CFP

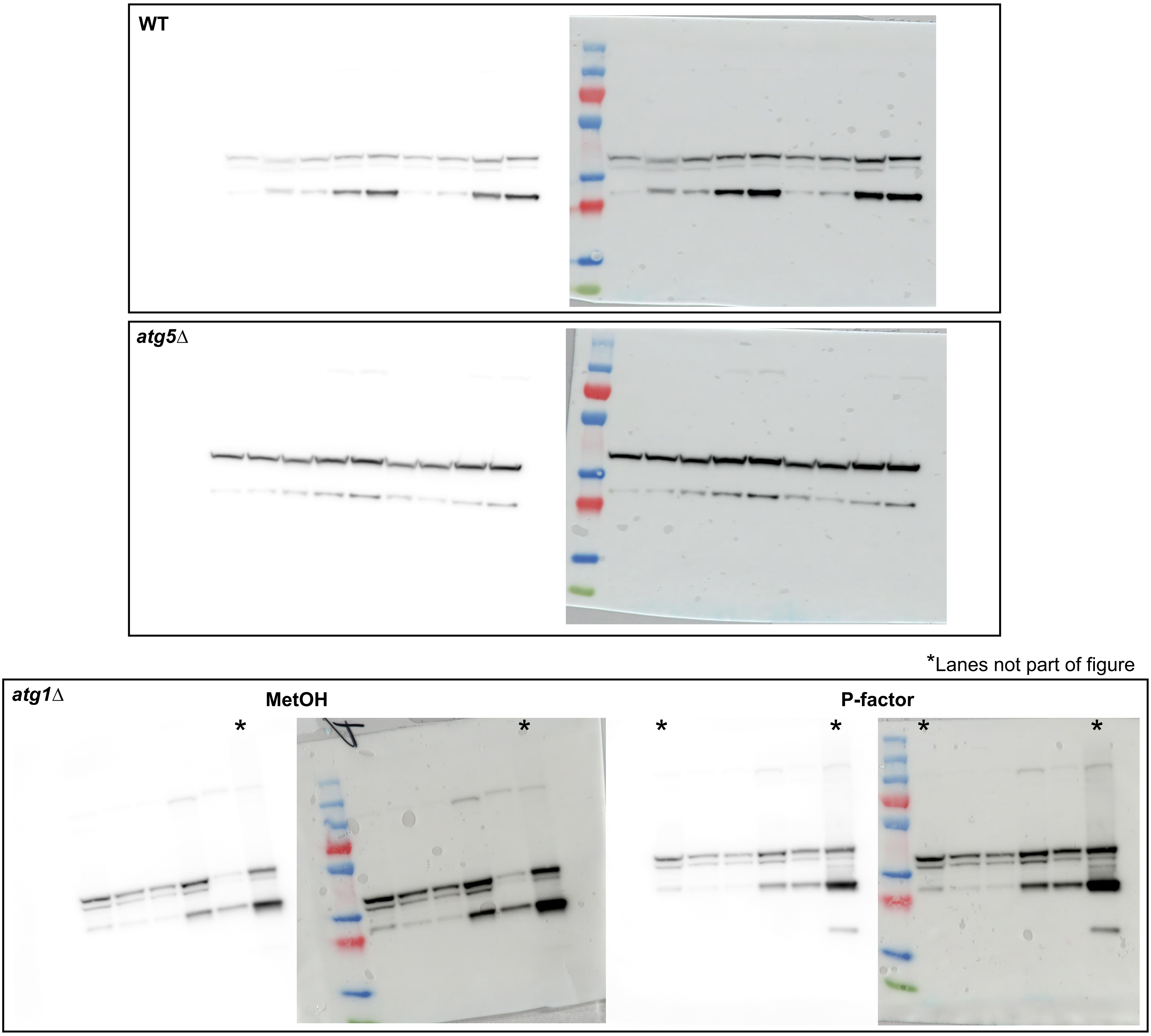

WT
atg5Δ
*Lanes not part of figure
atg1Δ
MetOH
P-factor
