# Supplementary material for: Proteomic and phosphoproteomic analyses reveal that TORC1 is reactivated by pheromone signaling during sexual reproduction in fission yeast
Source: PLoS Biol. 2024 Dec 20;22(12):e3002963. doi: 10.1371/journal.pbio.3002963 (PMC11750111; doi:10.1371/journal.pbio.3002963)
Supplement: Attachment — Submitted filename: 147794_1_rebuttal_3142026_sjlcbk.pdf [file pbio.3002963.s014.pdf]

Full Revision

Review COMMONS

Manuscript number: RC-2024-02543

Corresponding author(s): Sophie Martin

1. General Statements [optional]

The goal of study was to provide a first description of the phosphorylation changes that take place during the sexual reproduction of fission yeast cells, for which we developed protocols to synchronize the cell population (Figures 1 and 2) and obtained two high-quality datasets over sexual differentiation and cell-cell fusion (Figures 3 and 4). This led us to discover that TORC1 is re-activated by pheromone signaling during starvation, for which we show extensive validation (Figures 5 and 6) and probed the physiological meaning (Figure 7).

All three reviewers appreciated the work, and only raised minor criticisms, which we have fully addressed by text clarification and additional experiments. Reviewer 2 was the most critical, perhaps due to some lack of clarity on our part. We have strived to clarify the logic of the research in our text and provide more extensive data explanation. However, we also feel that some of the criticisms are unwarranted, and we explain below where we disagree with the reviewer or cannot provide the requested data comparison.

We provide as additional file a copy of the manuscript, where all changed text is shown in blue, so the modifications can be easily traced. New figure panels include 5F, S1A-B, S2 and S7D. Consequently, previous S1A became S1C and previous supplementary figures S2 to S6 have been renamed S3 to S7.

2. Point-by-point description of the revisions

Reviewer #1

Evidence, reproducibility and clarity

In this manuscript entitled "TORC1 reactivation by pheromone signaling revealed by phosphoproteomics of fission yeast sexual reproduction", the authors conducted detailed proteomic and phosphoproteomic analyses on fission yeast undergoing highly synchronous sexual differentiation. Their results demonstrate that TORC1 activity, which initially decreases at the onset of sexual differentiation, is later reactivated, a process crucial for the progression of sexual reproduction. Notably, this reactivation of TORC1 is triggered by pheromone stimulation, but is not dependent on autophagy.

Specific comments:
1 In Figure 5A, phosphorylation of Psk1 is observed 6 hr 15 min after starvation in homothallic cells

and h+ and h- cyclin deletion cells. Can the time course experiment show a decrease in phosphorylation followed by reactivation?

We have shown that heterothallic cells treated with P-factor show an initial decrease in P-Psk1 phosphorylation followed by re-phosphorylation (see Figure 5E). To probe whether the same dynamics is observed in mating mixtures, we have repeated this time course with the same time points in *h90* cells, which indeed shows an initial drop followed by re-phosphorylation. This is now shown as new Figure 5F.

2 In Figure 5C, the phosphorylation level of Psk1 in the S1837E mutant is as low as in the h- DMSO treatment, but Rps6 is phosphorylated. Is this phosphorylation of Rps6 Psk1-independent, differing from the results in Figure 5D?

This is a good question for which we can only speculate. The P-Rps6 signal is indeed increasing in the *tor2-S1837E* mutant whereas the P-Psk1 signal is not. P-Psk1 is a direct TORC1 target, whereas P-Rps6 is phosphorylated both downstream of Psk1 and downstream of TORC2-Gad8 (and perhaps also other kinases). Furthermore, there are complex cross-talks between TORC1 and TORC2. Thus, interpretation of the P-Rps6 signal is more difficult than the P-Psk1 signal. One possibility is that constitutive low TORC1 activity in the *tor2-S1837E* mutant leads to cell adaptation through some compensatory effect in signaling – for instance increase in TORC2 activity – which leads to the observed P-Rps6 signal. Such adaptation would likely not happen in *psk1Δ* cells, because these cells have no known phenotype (and therefore little adaptation pressure). It would also not happen upon acute TORC1 inhibition with rapamycin, which leads to loss of P-Rps6 as shown in Figure 5B.

3 The authors suggest that autophagy is not induced by pheromone signaling (page 10). However, in Figure 6B, the CFP signal, which is an autophagy marker, appears to be detected more strongly at 6 hours in the pheromone-treated samples compared to the control.

We have repeated this experiment several times, and the possible higher amount of cleaved CFP was not reproducible. We have exchanged the blot shown in the first version of the manuscript for one more representative of our observations. We also show a second replicate of this result in Figure S7D. We note that previous literature also concluded that there is no induction of autophagy during mating (Nakashima et al, Curr Genet 2006; Kohda et al, Genes to Cell 2007).

4 In Figure 6F and S6C, the phosphorylation dynamics of Psk1 appear to differ between wild type and atg1, atg5, and atg18a mutants. In atg1, re-phosphorylation induced by P-factor is weak, and in atg5 and atg18a, the decrease observed with control MeOH treatment appears to be weak.
Can autophagy be observed under the same conditions using CFP-Atg8 or other markers?

We agree that the dynamics are not identical between the mutants and the WT situation: compared to the data shown in Figure 5E, the downregulation of Psk1 phosphorylation is less marked in all cases, but also in WT, which is due to the presence of low levels glutamate in this experiment. However, in all cases, the early time point signals (20’ and 40’) are very similar between MetOH and P-factor treatments, whereas the late time point signals (240’ and 480’) are significantly higher in the P-factor treatment. In quantification of these ratio, we find a > 3 increase in *atg1Δ* and *atg5Δ* like in WT (as also detailed in response to comments from reviewer 3). Thus, the effect of P-factor signaling on promoting TORC1 activity does not require autophagy, even if availability of nitrogen is likely to modulate the quantitative outcome. We apologize that our text was a bit too cursive. We have now clarified this in results and discussion.

Regarding the question whether autophagy can be observed in the same conditions, we had shown in Figure 6D that autophagy is strongly decreased in *atg5Δ* and *atg18aΔ* but less so in *atg1Δ*, although in no case is CFP-Atg8 cleavage completely abrogated. We have now added blots showing CFP-Atg8 cleavage in each of the time courses in Figure S6C-D. These confirm the findings from Figure 6D that autophagy is reduced in *atg1Δ* and more reduced in *atg5Δ* and *atg18aΔ*, though some residual cleavage is still observed in these mutants.

5 The authors state that Rps6 phosphorylation is not important for mating (page 11). However, Figure 7A shows that the mating efficiency of the rps601 rps602AA strain is lower than that of the wild type, suggesting that phosphorylation of Rps602 plays a significant role in mating.

The mating efficiency of *rps601Δ rps602AA* is indeed lower than that of WT strains, but this reduction is completely accounted for by the *rps601* deletion, which indicates that mutation of the phosphorylation sites in Rps602 produces no additional phenotype. Hence our conclusion.

6 While inactivation of TORC1 in the temperature-sensitive tor2 mutant induces sexual differentiation, caffeine or auxin, which inhibits TORC1, decreases mating efficiency (Figure 7D). Caffeine and auxin do not act specifically on TORC1 alone, so it is possible that the decrease in TORC1 activity is not the sole cause of the reduced mating efficiency.
In relation to this, does the S1837E mutant with lower TORC1 activity show reduced mating efficiency as the deletion mutant of tco89?

We agree with this comment on auxin and caffein. We would have preferred to use rapamycin, which is very specific for TORC1, but could not for the reasons explained in the text. (Briefly: rapamycin is inhibitory to TORC1 in complex with Fkh1, but *fkh1Δ* is itself sterile, making any interpretation of the effect of rapamycin on TORC1 during sexual reproduction impossible.) We were not able to detect a reduction in overall mating efficiency of the *tor2-S1837E* mutant, despite early (agglutination) and late (spore viability) phenotypes. However, we believe that the array of evidence we provide is convincing that reduction of TORC1 activity compromises the sexual reproduction cycle of fission yeast.

Minor comments:
1 In Figure 4D, there is no legend explaining the colors in the graph.

Thanks – this is now corrected.

2 In Figure S6, the Psk1 blot is unclear.

We have repeated this western blot and now show a clearer one (now Figure S7A).

3 In Figure 7G, what do the colors and larger circles represent?

Sorry – colors represent individual experiment; dots individual datapoints; large circle average values per experiment. We have updated the legend with this information.

Significance

The work is sound and generally well-controlled. The data presented are novel and informative, and should be of interest to the readership. Specifically, the identification of pheromone-dependent

TORC1 reactivation provide significant insights into the regulatory pathways of yeast sexual differentiation.

We thank the reviewer for their comments and appreciation of our work. We hope that our revisions are satisfactory.

Reviewer #2

Evidence, reproducibility and clarity

In S. pombe cell-cell fusion during zygote formation is regulated by the Fus1 formin actin nucleator which forms an actin structure at the site of cell-cell fusionin a way that is coordinated with local release of enzymes that hydrolyze the cell walls at the site of attachment of paired cells to allow the plasma membranes to join together without cell lysis. The authors "aimed to describe protein phosphorylation changes during sexual differentiation and cell fusion in the fission yeast Schizosaccharomyces pombe". To do this they "synchronized cells pre-fusion and release them synchronously into the fusion process". This was done with a conditional fus1 (formin) allele which they thought blocks ells prefusion at restrictive conditions and synchronously promote fusion when reactivated. They made an optogenetic allele whether FUS1 N and C termini are expressed as separate polypeptides linked to A. thaliana photosensitive CIBN and CRY2 binding parners. They also performed a variety of other experiments to explore nitrogen starvation, autophagy and mating under different conditions and examine the prefusion to fusion transition in greater detail.

This paper describes a clever approach to try to enrich for cells that are prefusion using a blue light system with Fus1opto protein. The authors also intelligently use alternative sources of ammonia by shifting to glutamate and also delete 5 nonessential cyclins to increase the length of time S pombe is in G1 phase. However it is not clear that blocking Fus1 only acts at this step in the life cycle of S. pombe and data to confirm this are lacking (either published and or in this manuscript). In addition, it is not clear if the changes in growth conditions and cyclin background are permitting a physiologically relevant nitrogen starvation and mating although they do enhance ability to detect the elusive prefusion state. A discussion of what might be the difference with these changes in terms of proteomics and physiology is needed.

The phenotype of *fus1Δ* is well described in several previous papers (Petersen et al, 1995, 1998; Dudin et al, 2015; Billault-Chaumartin et al, 2022a, 2022b). The gene is only expressed as cells differentiate sexually. Deletion of *fus1* does not prevent sexual differentiation and very specifically blocks the fusion step. It produces no other known phenotype. We have added a sentence at the start of the results to state it and cited the most important prior work.

Regarding the changes in growth conditions (nitrogen source), the main difference between cells pre-grown in presence of ammonium or glutamate is the rate at which they mate, as shown in Figure 2. The final efficiency of both mating and fusion after 24h is very similar. We now show this new figure panels (Figure S1A-B). The same is true for the *cycΔ5* mutant (shown in the same panels). As a side note, these experimental conditions do not reveal or extend an elusive prefusion stage; on the contrary, they hasten the entry into differentiation such that there is better synchrony over the population.

We have included in the discussion a statement that these conditions have to be considered and that some phosphosites, notably regulating cell cycle progression due to the *cycΔ5* mutations, may be absent from our dataset.

This is a large body of work touching on many types of questions relating to mating in S pombe that will ultimately be a useful contribution to the field. Overall, the writing is fairly clear but needs tightening up with more explanation in the text. It is a bit difficult to follow in terms of flow and logic and comes across as a set of many different experiments not necessarily related to each other, rather than a story. Key take home points from the multiple phosphoproteomics experiments are not clear and their relationships to the other experiments in the paper are not clear. The table of values would be improved with a legend highlighting key values or some interpretation associated to understand what values are considered significant along with a sup figure that illustrates reproduciblilty of values and threshold cutoffs. Typically there are validation experiments for some of the values to confirm, this is absent.

We apologize if the flow of the paper was unclear to this reviewer. The logic of the work was simply to conduct phosphoproteomics discovery screens, which led to the surprising discovery that TORC1 is reactivated during starvation by pheromone signaling. The first two figures describe the methodological improvements for synchronization of cells necessary for the proteomics screens; the next two figures describe the screens; the last three figures focus on the phosphorylation of Rps6 discovered in our screens leading to our discovery of TORC1 reactivation and its physiological role. We agree that the description of the phosphoproteomics can be a bit dense, as there are lots of different observations that we put forward to both support the validity of the data and demonstrate the new findings. We chose to focus on one main finding from our screens – the phosphorylation of Rps6, which we extensively validated using phospho-specific antibodies, as shown in Figure 5. We further showed that the increase in phosphorylation was not specific to the *cycΔ5 fus1*^{opto} background used for cell synchronization, as it also happens in WT cells. Validation of other phosphorylation events awaits development of other phospho-specific antibodies.

Regarding Tables S1 and S2 containing all the phosphoproteomic data, we have increased readability in the following way:

- We expanded the first sheet that provides legend, listing the content of each tab and also including some information from the methods, in addition to explanation of the headers
- In particular, we added explicitly the significance thresholds that were used (which was described in the methods): i.e. for the starvation/mating time course, which has 3 biological replicates, changes were considered significant when the Benjamini-Hochberg adjusted p-value was < 0.05, for the cell fusion time course, which has 4 biological replicates, changes were considered significant at $p < 0.001$.
- We added to Table S2 the mating values without starvation subtraction.
- We organized the data with colors and headers to highlight the different columns and group the data in a logical way.

For clarity of which changes are significant: Table S1 provides a list of just these sites and proteins. In Table S2, which presents all the data, the corrected p-value can be used to sort according to significance. Any value larger than the threshold above is indicated as NS.

Regarding reproducibility, the dataset contains 3 (starvation/mating) or 4 biological replicates of the entire dataset. All the sites that show statistically significant changes are reproducible over the 3 or 4 replicates. To illustrate this visually, we have now put together a supplementary figure with examples of the similarity between replicates (new Figure S2A). We also kindly refer the reviewer to the website we constructed (https://pombephosphoproteomics.unige.ch/), where all individual traces with significant change can be visualized.

Additional points:
Indicate whether strains are isogenic in materials and methods or table s3

All *S. pombe* strains across all labs are isogenic.

Indicate references for method of protein extraction.

This is done. The method was adapted from Swaffer et al, Cell 2016.

Discuss what is known about light versus dark conditions on S pombe growth and mating and whether that impacts interpretation of data with fus1opto.

The *S. pombe* genome does not code for any light-sensing protein. Therefore, to our knowledge, there has not been a systematic study of growth in dark vs light condition. However, there have been several optogenetic studies (for instance: Lamas et al, PLoS Biol 2020; Gerganova et al, Sci Adv 2021; Sakai et al, Yeast 2024), where controls were performed, and which did not reveal any light-dependent growth issue. We have also performed the appropriate control of WT cells mating in light or dark conditions in this manuscript (see Figure 1D; 100% of WT cells fuse in both light and dark conditions).

The fusion efficiency calculation makes sense to this reviewer. However, it is not clear why a value of 2 is inserted in several of the other formulas such as mating efficiency and percent fusion on page 25,

This is to take into account that each zygote is formed by the fusion of two individual cells.

Fig1E. The experiment is lacking the all dark control. It is not clear why the changes in dark light are causing the changes in percent cell fusion since there is not so much difference between the relative time scales of dark and light outcomes. The critical window of blue light may be within the 18-24 hour window that currently has a wide spread and so one would want to titrate that window down to all dark and intermediatry amounts of light in this 6 hour period and more values at the 18 hr time point given the large spread. What amount of fusion is there with the fus1 null and the fus1opto kept in the dark 24 hr? Fig1A is striking to see the rapid changes at 4 minutes and 20 minutes after activation of Fus1opto. It appears the prefusion state is occurring at 0' based on cell body staining although the fus1opto is not yet activated. The experiment is lacking staining of nuclei to know where we are during zygote formation.

This panel 1E shows that, although the *fus1^{opto}* allele is very powerful on cells that have recently engaged in mating (as shown by the continuous light or the dark for 6h only, and also in Figure 1B-C), it becomes less efficient on “old” cell pairs.

The reason for the different success in cell fusion for cells held for different time in the dark is the following: As sexual differentiation starts, cells will progressively form cell pairs. In the light, Fus1^{opto} is active and paired cells immediately fuse. In the dark, they will stay in the paired pre-fusion state, as they are blocked by inactive Fus1^{opto}. Because of the asynchrony of sexual differentiation, a long dark incubation is required to accumulate a maximal number of cells as pre-fusion pairs. The critical observation is that fusion efficiency is reduced upon longer time spent in the dark (i.e. upon cells staying in the pre-fusion state for longer). This is not due to how much light was provided, as Fus1^{opto} requires only 20min of illumination to induce cell fusion, as pointed out by the reviewer and

shown in panels 1B-C. Instead, for currently unknown reasons, pre-fusion cell pairs eventually loose fusion competence. Therefore, *fus1*^{opto} will only be able to synchronize the process of cell fusion over the cell population if sexual differentiation is fast enough. We have clarified the text in the manuscript.

The data for *fus1*^{opto} cells continuously mated in the dark is shown in Figure 1D, which shows that these cells exhibit a baseline level of fusion at about 20%. The *fus1Δ* cells mated in the dark do not behave differently from those mated in the light and are completely unable to fuse.

We don't believe it is necessary to show nuclear staining, as we are monitoring cell-cell fusion (plasmogamy), which is the direct consequence of Fus1 activity, by visualizing the entry of in the M cell of cytosolic mTagBFP2 expressed only in the P cell.

Fig 2B It is not clear how the loss of the 5 cyclins leads to such tremendous long cell bodies and how this impacts the questions being asked.

The long shape of *cycΔ5* mutants is due to their elongated cell cycle (longer G1 phase). A longer cell size is a stereotypical consequence of cell cycle delay in *S. pombe*. This G1 delay facilitates sexual differentiation as cells are already in the pheromone-permissive phase of the cell cycle. It is possible that this genotype masks some phosphorylation changes that may normally occur in the WT cells for G1 arrest, a point we stated in the discussion.

Fig2B I would like to see images of the cells fusing without the cyclins to visualize the prefusion state in these grotesquely elongated cells. Are they forming normal cell-cell contacts, prefusion and zygotes? Not clear.

We are not sure what marker the reviewer would like to see in addition to what is already in Figure 2B. From the DIC images shown, we can easily distinguish pre-fusion pairs forming cell-cell contacts (but with cell wall at the contact site still present) from fused zygotes. We have now indicated with distinct colors the pre- and post-fusion states in this panel (orange and yellow dashed lines, respectively). We have also now provided a quantification of final mating and fusion efficiencies in this mutant background, which is not different from WT (new Figure S1A-B).

Fig2. Below Fig2B is a plot that is not described in figure legend and lacks a label. Should it be E and go with the description in D?. Looking at this plot I wonder what the results are for the control strain that has all of the cyclins.

This plot is indeed the quantification that goes with the description on the left. We have now relabeled as two distinct panels D and E, and clarified the legend.

The point of this plot is to show the synchrony we have obtained upon combining glutamate, *cycΔ5* and *fus1*^{opto}. If we were to perform the experiment in cells with all cyclins (but pre-grown in glutamate), there would be < 25% of cells engaged in mating after 3h (180min), as judged from the data shown on Figure 2A, which starts at 4h.

Figure 5 TORC1 reactivation by pheromone signaling. The authors use phosphorylation of Rps6 as a monitor for activation of Torc1. However, S6Kinase or RSK is activated by more than one signaling pathway including MAPK cascades that regulate mating. It is not clear that the reactivation they are monitoring is strictly the result of TORC1 reactivation.

We completely agree that Rps6 can be phosphorylated by several kinases, not all downstream of TORC1. In fission yeast, as clearly stated in our manuscript, Rps6 is phosphorylated by both Psk1

downstream of TORC1 and Gad8 downstream of TORC2. Our data very clearly show that the strong phosphorylation signal we see during mating is due to the TORC1-Psk1 branch, as it is abrogated by 1) rapamycin treatment, which is a specific TORC1 inhibitor, and 2) *psk1* deletion. We also show that Psk1, a direct TORC1 substrate, is itself re-phosphorylated during mating. Because Psk1 is a direct TORC1 substrate, we then use this phosphorylation event to monitor TORC1 activation in the rest of the manuscript. (Note that no peptide covering this phosphorylation site, whether phosphorylated or not, was identified in the MS data.) Thus, we are very confident of our conclusion that TORC1 is reactivated during sexual reproduction.

Figure 7 legend lacks information on E with the agar plates.

Figure 7E shows liquid cultures of WT and tor2-S1837E mutants, showing lack of cell aggregation in the mutant. We have added "liquid" to the panel for clarification.

SuppFig1 shows that fus1 fusogeni activity is small under wild type conditions. This raises the question of its importance. What is different in the altered conditions that enhance dependence on Fus1?

Fus1 is essential for cell fusion in all conditions. Figure S1 shows that the N-terminus of Fus1 (Fus1N), not full-length Fus1, has low fusogenic activity. More precisely, it shows that the low non-specific fusion we see in the *fus1*^{opto} strain in the dark (i.e. when Fus1N is not linked to the actin assembly function provided by Fus1C) is not a problem when sexual differentiation is rapid.

Supp Fig2 the yellow and pale blue writing difficult to discern. Better if it is black.
Supp Fig 3. It would help to match color for increase and decrease to be red and blue respectively in the GO term analysis to make it match the top part of the figure.

We have changed the color coding for Figures S2 to S5 to match with the heatmaps and remove the pale colors.

Supp Fig 4 Part E would have meaning if the authors were to connect the phospho sites with putative classes of kinases in another plot or table. How do these findings compare to other phosphoproteomic studies? There are Steve Gygi lab study and other studies on phosphoproteomics during pheromone signaling in s. cerevisiae for example. The study by these authors compares a variety of conditions. Which ones are used in Supp Fig 4E?

We have compared our dataset with all phosphoproteomic studies previously conducted in fission yeast (Cdc2, Orb6, TORC1/2). These analyses are shown in Figure 3D and 4D. From these kinases, Orb6 has a clear consensus site and Cdc2 (like MAPK) typically has proline-directed sites. Figure S4E simply looks at whether a simple logo emerges from our data as a whole or after removing the known Orb6 substrates and candidate CDK and MAPK substrates. The only point we are making here is that no consensus emerges, indicating that many kinases are at play. We can remove this panel if it is confusing.

We cannot easily compare our data to phosphoproteomic datasets in *S. cerevisiae* or other species, as phosphorylation sites tend to evolve rapidly and not be conserved in a one-to-one manner between species pairs (see for instance Studer et al, Science 2016), especially divergent ones such as *S. cerevisiae* and *S. pombe*. We note that the study from the Gygi lab the reviewer refers to (Li et al, J. Proteome Research 2007) did not perform a time course analysis, but only provides a snapshot of phosphorylation sites present upon pheromone treatment, where they identified 2288 sites in 985 proteins, of which we do not know which ones also exist without pheromone exposure.

We identified over 10’000 sites in 1884 proteins. These numbers cannot easily be compared, as it depends on the sensitivity of the mass-spectrometer.

Experiments to confirm the proteomic findings are lacking. The authors attempt to separate proteomic changes that relate to nitrogen starvation from other changes, for example mating. Their approach is to subtract the nitrogen starvation changes first and analyze what is left. This analysis reveals few changes for mating. First the actual list of changes and fold change for each protein or phosphosite should be referred to with highlight of any new phosphosite or proteins for the reader, likewise for nitrogen starvation rather than a vague summaries. Second, the outcome is very different from what has been found in S. cerevisiae where the induction conditions were specific to pheromone treatment of cells. It is possible that the authors are losing bona fide changes by subtracting the starvation response because both responses may have overlap. At a minimum this needs to be discussed in detail with references to what has already been published viz a viz mating and starvation responses in yeast.

We respectfully disagree with the reviewer about either statements that confirmation experiments are lacking or that we provided a vague summary. We provide all the data in Tables S1 and S2, of which we have now improved readability (see above). We also constructed a website through which all the data can be accessed (https://pombephosphoproteomics.unige.ch/). We are being completely transparent in data reporting. We have now also added the non-subtracted data in Table S2, as stated above.

Regarding data validation, we confirmed the Rps6 phosphorylation by using phospho-specific antibodies and extended this work to show reactivation of TORC1 by pheromone signaling in starvation conditions and physiological importance of this reactivation.

We stand by our strategy to subtract starvation data from the mating data, to extract mating-specific changes. The strategy, using linear regression analysis, will only remove sites for which the scale of changes is the same in both conditions. As stated in Figure 3C (second row), there is a substantial number of sites (156 sites in 95 proteins) that vary differently in starvation and mating, and these were retained. We now show some examples in Figure S2A to illustrate our strategy visually. We acknowledge that the subtraction strategy may lead to a slight underestimation of the true extent of changes, because it may in some cases increase the variation between replicates (due to addition of noise), which may reduce the statistical significance of a few sites. However, the enrichment in GO terms for the sites we identified as varying specifically during mating, and the identification of expected sites, both confirm the validity of the approach. We have added a couple of sentences in the methods to clarify this.

Finally, the reviewer states that the changes we observe are very different from those seen in *S. cerevisiae* and request a comparative analysis. In principle, it would not be very surprising if phosphorylation changes in *S. pombe* are different from those occurring in *S. cerevisiae*. These two organisms not only have a long evolutionary distance and thus phosphorylation events are likely to have evolved substantially. They also have very different physiologies, especially regarding sexual differentiation, as haploid *S. cerevisiae* cells mate in rich media conditions, whereas *S. pombe* mates only upon starvation. However, we find the request to perform comparative analysis with changes induced by pheromone in *S. cerevisiae* difficult, for two main reasons. First, as stated above, phosphorylation sites tend to evolve rapidly and not be conserved in a one-to-one manner between species pairs (see for instance Studer et al, Science 2016). Second, the literature does not contain equivalent data on even the number of changes upon pheromone treatment in *S. cerevisiae*. We found three studies with phosphoproteomic data of *S. cerevisiae* after pheromone exposure: One

early methodological study (Gruhler et al, MCP 2005) performed SILAC phosphoproteomics comparing pheromone-treated and untreated cells at a single time point and identified 139 changes from >700 phosphopeptides. However, this study is mainly methodological and shows a single biological sample. A second study (Li et al, J. Proteome Research 2007) identified existing phosphosites in pheromone-treated cells, but without performing a time course or even comparing to untreated cells, so it is not possible to know which of these sites are induced by pheromone. A third study performed extensive phosphoproteomics of pheromone and salt-treated cells to examine MAPK pathway crosstalk (Vaga et al, Mol Sys Biol 2014), but this study does not list their complete data. Thus, we find it difficult to even make a statement on whether the extent of changes is very different in *S. pombe* and *S. cerevisiae*.

A final concluding section of discussion that pulls things together is lacking from the manuscript.

In our discussion, we first discussed the datasets, what they revealed and how they will be useful for future investigations. Due to the extent of data, it is difficult to discuss it more without being repetitive with our text in the results section. Instead, we chose to then focus our discussion on the biological question we addressed, that of TORC1 reactivation. We are happy to hear about other suggestions.

In discussion paragraph 1 the authors discuss caveats and mention they found a couple of expected phosphorylation events. A table that puts together all known phosphorylation events t it hat is aligned with what was found in this study for mating and starvationautophagy induction should be included either in results or discussion to make the findings clear and relevant to what has been done.

We have now included a short table in Figure S2B listing the expected and identified increases in phosphorylation during sexual differentiation, which supports the description made in the results.

I believe the authors are able to address the concerns in a reasonable time frame and resubmit a modified manuscript that will be acceptable for publication. The length of time to address concerns is variable depending on what their needs are.

Significance

This is a large body of work touching on many types of questions relating to mating in S pombe that will ultimately be a useful contribution to the field. Overall, the writing is fairly clear but needs tightening up with more explanation in the text. It is a bit difficult to follow in terms of flow and logic and comes across as a set of many different experiments not necessarily related to each other, rather than a story. Key take home points from the multiple phosphoproteomics experiments are not clear and their relationships to the other experiments in the paper are not clear. The table of values would be improved with a legend highlighting key values or some interpretation associated to understand what values are considered significant along with a sup figure that illustrates reproduciblilty of values and threshold cutoffs. Typically there are validation experiments for some of the values to confirm, this is absent.

We thank the reviewer for their comments on our work. We hope we have convinced them that the data we present are highly reproducible and that the work we performed on TORC1 represents an important validation of the screen. Our aim was to present not only a robust dataset, but also a significant advance on our biological understanding of the mating process.

Reviewer #3

Evidence, reproducibility and clarity

The manuscript by Melvin Bérard et al. describes total and phosphoproteome changes that occur during specific stages of sexual reproduction in the fission yeast Schizosaccharomyces pombe and demonstrates an unexpected role for TORC1 during this process. The authors designed a strain and procedure for robust and efficient synchronization of haploid cell mating, allowing the analysis of large population of cells, which was a challenging prerequisite for biochemistry and proteomics. While the effects of pheromone signaling and cell fusion have been extensively characterized at the genetic and cellular levels, much less was known about the dynamics of protein phosphorylation during mating. Therefore, the optogenetic tool, culture conditions, and the rich phospho-proteomic dataset reported here represent valuable resources for the fission yeast community. Mass spectrometry data are shown in a format that makes data mining quick and easy, including a well-designed website.

This work allows digging into the signaling cues and mechanisms that drive the early stages of fission yeast sexual differentiation, with many new and unexpected observations. Amongst these, phosphorylation of one of the canonical TORC1 substrate, S6, is shown to increase several fold during mating, contrasting with the well-known inactivation of TORC1 activity upon nutrient starvation. Biochemical and genetic evidence demonstrate that TORC1 is responsible for the mating-induced increase of S6 phosphorylation, through phosphorylation of Psk1, as previously shown in proliferating conditions. Altogether, the authors suggest a model by which TORC1 is first inactivated in response to the lack of nitrogen to promote catabolic processes, such as autophagy, and then re-activated at later stages to promote mating, meiosis and sporulation.

Overall, the experiments are well conducted, properly described and the results well interpreted except in a few cases (see major issues below). The manuscript is pleasant to read and very well presented. However, I have several concerns that need to be addressed before considering it for publication in any journal.

Major issues:

- The introduction overlooks several studies that already characterized the effects of signaling pathways and the roles of their substrates during S. pombe sexual differentiation, giving the false impression that there is "extremely limited knowledge" about this process. Without listing them all , here are a few studies that should be mentioned in the introduction: the cAMP-PKA pathway regulates mating through Rst2 phosphorylation (10.1128/MCB.22.1.1-11.2002); AMPK controls sexual differentiation through phosphorylation of Ssp1 (10.1242/jcs.098533); upstream regulators of TORC1 are crucial regulators of mating (eg 10.1093/genetics/155.2.611 and 10.1534/genetics.109.105379 for Rheb; 10.1242/jcs.094219 for Rag); downstream effectors phosphorylated either directly or indirectly by TORC1 and TORC2 with a role in mating (10.1093/emboj/cdg298 for Gad8, Otsubo et al, 2014 and 2018, which are cited only later in the manuscript, 10.15252/embr.201744942 for SAGA, 10.26508/lsa.202101082 for Gsk3-Pub1); Cdc2 controls sexual differentiation through Fkh2 phosphorylation (10.1038/sj.emboj.7601949). Even the nitrogen stress and TORC1/2 phosphoproteome studies of Halova et al 2021 is only cited later in the manuscript. The introduction should be rewritten to include these results.

Our intention was not to mis-represent the previous knowledge of the signaling of sexual differentiation, for which there is indeed an appreciable amount in *S. pombe*. We had cited some of

these papers later in the manuscript, simply because we felt that it made the reading easier to not introduce all information about TOR signaling at first, but bring some of this knowledge at the point of the paper when it is needed. We have extended our introduction to include some more information on known signaling and phosphorylation events, including papers mentioned above. We did not include all of them, as we think that an exhaustive description is not necessary to introduce the global picture at the start of the study. However, most of these studies are cited at some point in our manuscript, which with 129 citations is already covering a vast amount of previous literature.

We have also reworded the sentence cited by the reviewer to "While many kinases (and some phosphatases) have well established roles in promoting sexual differentiation and cell fusion, we still have limited knowledge about their substrates".

- The authors mention that the expression and phosphorylation of meiotic and sporulation factors occurs ahead of gamete fusion, but it is not clear which expression data they refer to. In addition, the mating and fusion counts shown in Figure 2D suggest that there might be a significant amount of cells that mated before the 150+30 minute time point. Moreover, the ~20% leakage of the fus1 optogenetic allele might contribute a significant amount of mated cells at the start of the time-course. It is thus important to count the number of mated and fused cells in a time-course experiment that covers the same 45 minute intervals used for proteomic/phospho-proteomic analyses shown in Figure 3. Even a relatively small fraction of cells already engaged into meiosis might explain the early expression of meiotic/sporulation genes.

We apologize, as this comment likely stems from lack of clarity in the manuscript. We agree that it is important to control that there is no cell fusion in the conditions used for the (phospho)proteomic time course during mating, to be sure that that the phosphorylation of meiotic and sporulation factors we observed really happened before fusion. In Figure S1, we show the requested quantification for cells treated exactly as those collected for proteomics, i.e. after cells were held separately 2h in the dark and then mixed for 2h30. This corresponds to 150 min in the scheme shown in Figure 3A (between the 135 and 180 min time points). At that time point, we see no fusion at all in 2 of 3 experiments, and < 5% in the third repeat. Thus, we can safely conclude that the increase in phosphorylation we see for Mis4, Rec10, Rec8, Spo5 and other factors, which is extremely reproducible between replicates (please consult https://pombephosphoproteomics.unige.ch/) and already very clear at 135 min, is not due to leakage fusion.

The almost 20% fusion induced by the inactive *fus1^{opto}* allele (due to expression of Fus1 N-terminus) reported in Figure 1D and S1 (left bar), are late events.

We have added mention of Figure S1 in the result section where we describe phosphorylation of the meiosis and sporulation factors and clarified the legend of Figure S1.

- Although several experiments were performed to characterize the regulation of Rps6 (figure 5) and Psk1 (Figure 6) phosphorylation, it is unclear whether all effects are reproducible and which experiments were replicated. This is important to show a semi-quantitative estimation of the effects of culture conditions / mutations on this modification on a graph, rather than single Western blot experiments. For example, nitrogen starved h- cells show different levels of S6 phosphorylation when comparing Figure 5A and 5C. In figure 6F, the dynamics of Psk1 phosphorylation appears different between WT, atg1Δ, and atg5Δ strains treated with P-factor, contrasting with the authors' conclusion that pheromone dependent TORC1 re-activation does not require the autophagy pathway. Finally, phosphorylation should be normalized to total levels of the corresponding protein. For example, Rps6 total levels vary significantly between conditions and strains in Figure 5A.

Every western blot experiment shown has been confirmed at least twice from independent biological replicates. Obtaining even semi-quantitative values from western blots is very difficult, due to different signal intensity for each blot (notably because of variations in antibody quality, for instance due to re-use of antibody solution to economize use, and exposure times). As pointed out by the reviewer, quantifications of phosphorylation levels should be normalized to total levels of the corresponding protein, but this will yield very different outcomes between blots, for instance depending on blot exposure time. To correct for blot-to-blot variations in signal intensity, the only valid quantifications are ratios, where we additionally normalize the ratio of P-Psk1/Psk1 (or P-Rps6/Rps6) for a strain/condition of interest to a control strain/condition on the same blot. Using this method, we measured the fold increase in the P-Psk1/Psk1 and P-Rps6/Rps6 signal in *h90* strains in -N conditions, normalizing to the corresponding signal in *h-* strains, which is > 3.3 in all experiment (7 independent experiments for P-Rps6; 2 independent experiments for P-Rps6 in the *cycΔ5* background; 3 independent experiments for P-Psk1). However, even this value varies hugely, largely dependent on how strong the phosphorylation signal is in the *h-* strain (if there is almost no band, the denominator is close to zero, leading to a very large and likely inaccurate ratio; values range from 3.3 to 273.4). For this reason, we prefer not to provide numbers, which we feel may give a false sense of exact quantity. In any case, these numbers are now available in this response, which will be openly available.

Using the same method, we have quantified the drop in P-Rps6/Rps6 and P-Psk1/Psk1 levels upon rapamycin, which ranges from 2.5 to 10-fold (in 3 independent experiments each), the absence of effect of rapamycin in the *tor2-S1837E* mutant (1.2 to 1.3-fold change only; 2 experiments), the drop in P-Rps6/Rps6 in *psk1Δ* (10 to 20-fold in 4 independent experiments), and the effect of P-factor vs DMSO, which leads to a > 9-fold increase in P-Rps6/Rps6 and > 6-fold increase in P-Psk1/Psk1 after 240 min (2 and 3 independent experiments, respectively).

Regarding the effect of autophagy, we agree that the dynamics are not identical to those in WT. However, in all autophagy mutants, the P-Psk1 is initially reduced upon starvation. It stays low at late time points in control conditions (MetOH), but re-increases in P-factor treated cells. Quantification of the P-Psk1/Psk1 signal at the 240 or 480 time points in P-factor treated cells normalized to the same time point in MetOH-treated cells gives values > 3 in *atg1Δ* and *atg5Δ* like in WT. The *atg18aΔ* value is closer to 2. Thus, we feel it is correct to conclude that the effect of P-factor signaling on promoting TORC1 activity does not require autophagy, even if availability of nitrogen is likely to modulate the precise outcome. We apologize that our text was a bit too cursive. We have now clarified this in results and discussion.

- The differential effects observed in leucine auxotrophs could be due to lower TORC1 activity, as suggested by the authors, or, rather by higher TORC1 activity caused by the supplementation of media with leucine during exponential growth before nitrogen starvation (which is in fact both a nitrogen and leucine starvation). For this, one simple experiment is to measure the change of P-Rps6 and P-Psk1 levels in rich versus starved media of leucine prototrophs and auxotrophs.

Based on the low P-Psk1 signal in leucine auxotrophs during sexual reproduction, we have proposed that these cells have <u>low</u> TORC1 activity. The reviewer suggests the alternative hypothesis that these cells may have <u>higher</u> TORC1 activity during exponential growth due to leucine supplementation. In our view, several lines of evidence concur to make this alternative hypothesis unlikely.

First, although the response of cells to leucine starvation is admittedly complex, the interpretation of prior evidence was that leucine auxotrophs have <u>low</u> TORC1 activity during exponential growth (discussed in Ohtsuka et al, FEMS Yeast Research 2022). Notable previous evidence is that

leucine auxotrophs but not WT cells are sensitive to rapamycin during exponential growth (Weisman et al, Genetics 2005) and show fast onset of autophagy upon leucine starvation (Corral-Ramos et al, Autophagy 2022). In these published data, cells were (pre-)grown with leucine supplementation. In our own data, all experiments were also performed on cells pre-grown in MSL + amino acids, making it unlikely that supplementation causes the observed difference in TORC1 activity between WT and leucine auxotrophs.

Second, because starvation first induces a drop in P-Rps6 and P-Psk1 levels before pheromone signaling promotes their re-increase at later time points, it seems more difficult to imagine how a potential change in the pre-starvation levels of TORC1 activity (especially in the other direction to the one observed during sexual differentiation) would affect these late post-starvation time points.

To conclusively examine this point, we have compared P-Psk1 levels in WT and leucine auxotrophs during exponential growth (both grown with leucine supplementation) (Reviewer Figure 1). This experiment shows slightly higher P-Psk1 signal in the prototrophic cells, as we observed in our starvation/mating samples. Thus, we conclude that cells incapable of synthetizing their own leucine exhibit lower TORC1 signaling, even when provided with environmental leucine.

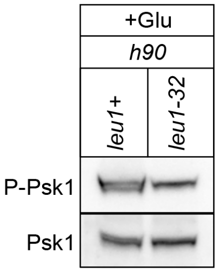

Reviewer Fig 1. Phospho-Psk1 levels in cells during exponential growth. Cells were grown in MSL + glutamate, supplemented with leucine.

Minor comments:

- Can the authors please clarify what we are looking at in Figure 1B. It looks like haploid cells are already fused before light activation (0' time point) in the movie snapshots shown with the yellow contour.

The contour shows the cell pair, which is initially pre-fusion as shown from the P-cell-restricted fluorescence of the Pmap3-mtag-BFP2 cytosolic marker. We have updated the colors of the cell pair contour, which we show in orange for pre-fusion pairs and in yellow for post-fusion ones (as done in Figure 2B). This is now specified in the legends.

- It is unclear why the authors decided to not wait longer in the dark (eg 12 hours - Figure 1E), in order to get more mating cells. The effect on the percentage of cell fusion does not appear so strong. Related to this graph, an ANOVA should be performed to compare each condition, rather than a t-test, because n=more than two sample means are analyzed altogether.

The reasons are twofold. First, at 12h, the drop in fusion competence is indeed still modest, but in the conditions used in Figure 1E, the plateau of cells mating has not yet been reached at this time point. Second, getting more synchronous sexual differentiation allowed us to obtain much better data about this process. Our guess is that, if we had done the mating time course of Figure 3 over 12h, we would not have obtained as clear dynamics of phosphorylation changes. We have replaced the t-test by an ANOVA, which is indeed more appropriate in this case.

- I recommend to use different colors/labels for the mating and starvation samples shown in the PCA analyses of Figs. 3B and 4B, to better differentiate between the two conditions.

We have changed the symbols in Figure 3B to differentiate the mating (closed symbols) and starvation (open symbols) samples. In Figure 4B, there is no confusion as there is only one time

course through cell-cell fusion.

- There are many interesting observations and I don't expect the authors to discuss them all. However, given the focus of the manuscript on TOR signaling, I recommend to discuss the changes in TORC2 substrate phosphorylation, many of which appear to decrease upon starvation, mating, and fusion, contrasting with the prevailing idea that TORC2 is activated (or at least required) during sexual differentiation.

Our data indeed suggest that there isn't a massive increase in TORC2 activity during mating, as judged from the observation that there is no significant increase in phosphorylated Gad8 in h90 vs h- cells in -N conditions (see Figure 5B). In agreement with this, we see no significant enrichment of TORC2 substrates during mating in either the group of increasing or decreasing phosphosites, although TORC2 substrates appear enriched in the phosphosites that decrease upon starvation (see Figure 3D). These observations are consistent with the well-established notion that TORC2 is required for sexual differentiation but suggest it may not be specifically activated. During cell-cell fusion, we see a potentially interesting enrichment of TORC2 targets in phosphosites that increase, but only when using data from one of the two published TOR phosphoproteomes (Mak et al, EMBO J 2021).

We have added one sentence to the discussion, stating "Our data also suggest that the activity TORC2, although it is essential for fertility, may not increase during sexual reproduction, as observed from the observation that Gad8 phosphorylation does not increase".

- Why is there no apparent cleavage of CFP-Atg8 in control conditions (MetOH) upon a shift to low nitrogen medium (Figure 6B)?

We have replaced this blot by a new one following this comment and comment #3 from reviewer 1. We are not exactly sure to which time point the reviewer refers in their comment. Cleavage of CFP-Atg8 happens progressively over a few hours in low N conditions. The cleaved band becomes stronger at the 240 min time point. In some cultures, we have seen a small amount of cleaved CFP in cells grown in medium +N (glutamate), which likely reflects historical accumulation of CFP in the vacuole.

Significance

This work allows digging into the signaling cues and mechanisms that drive the early stages of fission yeast sexual differentiation, with many new and unexpected observations.

Overall, the experiments are well conducted, properly described and the results well interpreted except in a few cases (see major issues below). The manuscript is pleasant to read and very well presented. However, I have several concerns that need to be addressed before considering it for publication in any journal.

We thank the reviewer for their careful assessment and appreciation of our work. We hope that our responses, clarifications and new experiments are satisfactory.
