# Supplementary material for: Proteomic and phosphoproteomic analyses reveal that TORC1 is reactivated by pheromone signaling during sexual reproduction in fission yeast
Source: PLoS Biol. 2024 Dec 20;22(12):e3002963. doi: 10.1371/journal.pbio.3002963 (PMC11750111; doi:10.1371/journal.pbio.3002963)
Supplement: Attachment — Submitted filename: Response to reviewers.docx [file pbio.3002963.s016.docx]

We thank the reviewer for their second reading of our work and their interest.

Reviewer #1: In this manuscript, the authors explore phosphoproteomic changes in the fission yeast S. pombe during highly synchronous sexual differentiation, highlighting a significant role for TORC1. The authors have adequately addressed the previous review's comments, resulting in a much improved manuscript.

Thank you!


Reviewer #2: This manuscript has satisfied this reviewer's comments in their response. I think it is worthy of acceptance. That said, I believe that several minor changes to the text would be an improvement. These are not required for acceptance

Thank you! We have made small modification in response to the comments below.

1.Reviewer comment on response to reviewer 1:
2 In Figure 5C, the phosphorylation level of Psk1 in the S1837E mutant is as low as in the h- DMSO treatment, but Rps6 is phosphorylated. Is this phosphorylation of Rps6 Psk1-independent, differing from the results in Figure 5D? 
This is a good question for which we can only speculate. The P-Rps6 signal is indeed increasing in the tor2-S1837E mutant whereas the P-Psk1 signal is not. P-Psk1 is a direct TORC1 target, whereas P-Rps6 is phosphorylated both downstream of Psk1 and downstream of TORC2-Gad8 (and perhaps also other kinases). Furthermore, there are complex cross-talks between TORC1 and TORC2. Thus, interpretation of the P-Rps6 signal is more difficult than the P-Psk1 signal. One possibility is that constitutive low TORC1 activity in the tor2-S1837E mutant leads to cell adaptation through some compensatory effect in signaling - for instance increase in TORC2 activity - which leads to the observed P-Rps6 signal. Such adaptation would likely not happen in psk1∆ cells, because these cells have no known phenotype (and therefore little adaptation pressure). It would also not happen upon acute TORC1 inhibition with rapamycin, which leads to loss of P-Rps6 as shown in Figure 5B. 
This reviewer thinks the authors response here is on target and should be a part of the discussion. They only do one experiment to support the discussion and it might be something to raise as an outstanding question.

We added the following sentence to the discussion:

We note however that pheromone-dependent phosphorylation of Rps6 (but not Psk1) is still observed in the hypomorphic *tor2-S1837E* mutant, suggesting contribution of other kinases than Psk1 to Rps6 phosphorylation, perhaps as cellular adaptation to constitutive reduction of TORC1 activity.

2. This reviewer is commenting on the response below to reviewer 1:
3 The authors suggest that autophagy is not induced by pheromone signaling (page 10). However, in Figure 6B, the CFP signal, which is an autophagy marker, appears to be detected more strongly at 6 hours in the pheromone-treated samples compared to the control.

We have repeated this experiment several times, and the possible higher amount of cleaved CFP was not reproducible. We have exchanged the blot shown in the first version of the manuscript for one more representative of our observations. We also show a second replicate of this result in Figure S7D. We note that previous literature also concluded that there is no induction of autophagy during mating (Nakashima et al, Curr Genet 2006; Kohda et al, Genes to Cell 2007). 

Author text carefully explains:
"We first used these conditions to probe whether starvation-dependent induction of autophagy is modulated by pheromone signaling. As a readout, we used CFP-Atg8 cleavage, as CFP-Atg8 is delivered to vacuoles by the autophagic process, where only the CFP moiety is resistant to vacuolar proteases. In WT cells, CFP-Atg8 cleavage occurred upon transfer to low-glutamate conditions and increased over time in a similar manner whether cells were treated with MetOH or P-factor (Fig 6B). This suggests that pheromone signaling does not induce autophagy. When we probed CFP-Atg8 cleavage in atg1∆ as control, we were surprised to observe significant residual levels of free CFP (Figure 6C, S7B), as deletion of this upstream regulatory kinase is reported to fully block autophagy (Mukaiyama et al., 2009; Pan et al., 2020; Sun et al., 2013). We reasoned that this difference may be due to differences in media or nitrogen source, as we used MSL + glutamate, while earlier studies used EMM + ammonium. Indeed, whereas exchanging glutamate for ammonium during growth before starvation had no effect on the observed cleavage in MSL medium (Figure S7B), using EMM medium abrogated cleavage (Figure 6C), as previously reported. Thus, for currently unknown reasons, deletion of Atg1 kinase only partially prevents autophagy in MSL medium. "

This reviewer thinks the discussion needs better summary relating to the differences with glutamate versus other nitrogen sources as well as listing of the use of glutamate instead of ammonium and the type of medium in their caveat paragraph.

We have added to the discussion the following sentence:

[Our finding should invite examination of the role of medium composition on autophagy] and more generally on cell physiology. All our experiments, except where indicated, were conducted upon starvation from glutamate-containing medium.

3. Request for changes to the caveat paragraph in Discussion: I paste the paragraph below and insert suggested changes as a guide to the authors in bold font with brackets so the authors can understand what this reviewer thinks needs to be included in the paragraph.

First, the cyc5∆ cells lacking all [FIVE] non-essential cyclins [i.e. cig1∆ cig2∆ crs1∆ puc1∆ rem1∆ ], which extends the differentiation-permissive G1 phase [and greatly increases cell length], may mask some of the approach and suggest that identified phosphorylation events also occur in wildtype strains. Third, we used glutamate instead of ammonium to hasten differentiation which may affect the physiological response in unknown ways and discovered that the medium (i.e. MSL versus EMM) influences signaling outcome  (see discussion of Figures 6 and S7B in results)."""

"""We have set up robust synchronization procedures to conduct population-based assays of mating and fusing cells, which will be of general use for other -omics analyses. It is however important to bear in mind the specific conditions allowing optimal synchrony. First, the cyc5∆ cells lacking all [FIVE] non-essential cyclins [i.e. cig1∆ cig2∆ crs1∆ puc1∆ rem1∆ ], which extends the differentiation-permissive G1 phase [and greatly increases cell length], may mask some of the (phospho-)proteomic changes governing the starvation- and/or pheromone-dependent cell cycle arrest of wildtype cells (Stern and Nurse, 1997; Stern and Nurse, 1998). Second, because of the use of the split fus1opto allele for synchronization during cell-cell fusion, we should be careful about interpretation of phosphorylation events on Fus1 and accessory proteins, as these may not represent native changes at the fusion site. Nevertheless, the few phosphorylation events that were expected (for instance that
of the MAPK Spk1 active site; (Kelsall et al., 2019)) or independently confirmed here (Rps6) validate the approach and suggest that identified phosphorylation events also occur in wildtype strains. Third, we used glutamate instead of ammonium to hasten differentiation which may affect the physiological response in unknown ways and found evidence that the type of medium (i.e. MSL versus EMM) also may influence signaling outcome  (see discussion of Figures 6 and S7B in results)."

We have added the details shown in brackets above ([FIVE] non-essential cyclins [cig1∆ cig2∆ crs1∆ puc1∆ rem1∆]) and also added the following sentences to that paragraph:

Third, we have used glutamate in MSL medium, instead of ammonium for pre-growth of cells to hasten differentiation, and found evidence that medium composition (MSL vs. EMM) may influence signaling outcome (see discussion on autophagy below).

3. With respect to discussion about phospho-proteomics presentation in Figure S5E.  The author appropriately responded to reviewer comments. In response to author question about S5E cluster motifs, this reviewer thinks E could be enlarged to make it more readable rather than removed but this is not essential.

We have not enlarged this panel, simply for space considerations in the figure. It is high resolution, so readers can zoom in the pdf to see details if required.

4. With respect to the choice of subtracting starvation data to reveal mating specific data. As the authors correctly wrote in the first sentence of their abstract: "Starvation, which is associated with inactivation of the growth-promoting TOR complex 1 (TORC1), is a strong environmental signal for cell differentiation." 
The authors have the right to present the data as they wish and subtract out the starvation data. However, starvation induced events may be involved in some of the mating response which is induced under conditions of nitrogen starvation in the presence of cells of opposite mating type. In addition, in their phosphor-proteomic analysis they did not uncover all expected phosphorylation sites for mating which leads one to wonder if any were removed during their subtraction. If this was checked it should be stated in the results section or methods section.

We answered this point in our previous response. For expected targets, we also looked at the uncorrected data. Some sites are simply missing from any MS analysis.

5. Figure S2B is a positive addition. A sentence that explains clearly that some but not all expected sites were found along with new ones should be included in its presentation. It is worth considering whether the missing ones found in the starvation phospho-proteomic data that was subtracted out and if not that might also be mentioned.

We feel the figure panel and its legend, as well as the description in the results (2^nd^ paragraph of page 8), are quite clear that we did not recover all expected sites. Any MS data is incomplete – it is difficult to make a stronger statement about absent data.

Reviewer #3: I have now gone through the manuscript and appreciate the efforts made to address my concerns. The authors provided additional data and clarifications that strengthen the manuscript and make it suitable for publication.
Of note, I understand why the authors feel uncomfortable about quantifying Western blots, but I think that one simple way to get an estimation is to load for example 3 lanes with decreasing quantities of one extract to give an idea of the range of signal variation that is detectable on a blot. Nevertheless, the key point of reproducibility was satisfactorily addressed.

Thank you!
